# The ancient evolutionary origins of Scleractinia revealed by azooxanthellate corals

**DOI:** 10.1186/1471-2148-11-316

**Published:** 2011-10-28

**Authors:** Jarosław Stolarski, Marcelo V Kitahara, David J Miller, Stephen D Cairns, Maciej Mazur, Anders Meibom

**Affiliations:** 1 Institute of Paleobiology, Polish Academy of Sciences, Twarda 51/55, PL-00-818 Warsaw, Poland; 2 ARC Centre of Excellence for Coral Reef Studies and Coral Genomics Group, James Cook University, Townsville, QLD 4811, Australia; 3 Department of Invertebrate Zoology, National Museum of Natural History, Smithsonian Institution, Washington, D.C., 20560 USA; 4 Department of Chemistry, Laboratory of Electrochemistry, University of Warsaw, Pasteura 1, PL-02-093 Warsaw, Poland; 5 Muséum National d'Histoire Naturelle, Laboratoire de Mineralogie et Cosmochimie du Museum, LMCM UMR 7202, Case Postale 52, 61 rue Buffon, 75005 Paris, France

## Abstract

**Background:**

Scleractinian corals are currently a focus of major interest because of their ecological importance and the uncertain fate of coral reefs in the face of increasing anthropogenic pressure. Despite this, remarkably little is known about the evolutionary origins of corals. The Scleractinia suddenly appear in the fossil record about 240 Ma, but the range of morphological variation seen in these Middle Triassic fossils is comparable to that of modern scleractinians, implying much earlier origins that have so far remained elusive. A significant weakness in reconstruction(s) of early coral evolution is that deep-sea corals have been poorly represented in molecular phylogenetic analyses.

**Results:**

By adding new data from a large and representative range of deep-water species to existing molecular datasets and applying a relaxed molecular clock, we show that two exclusively deep-sea families, the Gardineriidae and Micrabaciidae, diverged prior to the Complexa/Robusta coral split around 425 Ma, thereby pushing the evolutionary origin of scleractinian corals deep into the Paleozoic.

**Conclusions:**

The early divergence and distinctive morphologies of the extant gardineriid and micrabaciid corals suggest a link with Ordovician "scleractiniamorph" fossils that were previously assumed to represent extinct anthozoan skeletonized lineages. Therefore, scleractinian corals most likely evolved from Paleozoic soft-bodied ancestors. Modern shallow-water Scleractinia, which are dependent on symbionts, appear to have had several independent origins from solitary, non-symbiotic precursors. The Scleractinia have survived periods of massive climate change in the past, suggesting that as a lineage they may be less vulnerable to future changes than often assumed.

## Background

The two most popular hypotheses put forward to account for scleractinian origins are that they are either descendants of late Paleozoic rugose corals that survived the mass extinction at the Permian/Triassic boundary [1-3] or, that they evolved from soft-bodied (corallimorpharian-like) ancestors by gaining the ability to deposit a calcified skeleton [4-6]. Difficulties with the former hypothesis include that it requires major changes in both the composition of the skeleton, which was calcite in the case of Rugosa, but is aragonite in Scleractinia, and the symmetry of septal insertion [4], characters that are otherwise highly conserved. By contrast with Rugosa, some Permian fossils (known as scleractiniamorphs) appear to have had aragonite skeletons (*Numidiaphyllum*, *Houchnagocyathus*) and may be the immediate ancestors of some Triassic scleractinian coral lineages [7,8]. Intriguingly, some early Paleozoic "scleractiniamorphs" (kilbuchophyllids from the Ordovician, ca. 450 Mya) have patterns of septal insertion that are indistinguishable from that of modern corals [9,10], suggesting that these could represent the very early scleractinians. However, one objection to this idea has been the long time-gap separating the two groups in the fossil record.

Beyond implying that most extant scleractinians fall into two major clades (Robusta and Complexa) that are assumed to have diverged in the Late Carboniferous, ca. 300 Ma [11,12], molecular data have so far not added significantly to our understanding of early coral evolution. One reason for this may be that molecular phylogenetics has focused primarily on shallow-water corals, most of which harbor symbiotic dinoflagellates commonly known as zooxanthellae, whereas azooxanthellate, deep-water corals that account for approximately half of extant scleractinian species, have largely been ignored in these analyses [13]. The few studies that have included sequences from azooxanthellate scleractinians have led to conflicting interpretations of scleractinian phylogeny. For example, the phylogenetic reconstruction based on mitochondrial (12S rDNA) and nuclear (partial 28S rDNA) data for 80 scleractinian species (18 of which were azooxanthellate) suggested that all azooxanthellate, deep-water lineages originated from symbiotic, shallow-water ancestors [14]. In contrast, another study based on COX1 [15] found that members of the Gardineriidae and Micrabaciidae families formed a deeply diverging clade that may represent the oldest extant scleractinian lineage and that modern deep-water species diverge at or near the bases of both the Robusta and Complexa, implying that the evolutionary origin of scleractinians is best sought in deep-water rather than shallow-water (primarily zooxanthellate) coral species. These contradictory interpretations motivated us to extend phylogenetic analyses of a large and representative range of deep and shallow water corals (more than 10% of all extant deep-sea species; see Additional file 1) beyond COX1, to include data for the mitochondrial 12S and 16S rDNAs, and the nuclear 28S rDNA, in an attempt to clarify scleractinian origins and relationships. In addition, the ages of the major scleractinian lineages were estimated, and the origins of the Order explored. The divergence time estimates generated here bridge the gap with fossils, allowing the integration of the morphologically similar Paleozoic "scleractiniamorphs" into Scleractinia.

## Results and Discussion

**Figure 1:**
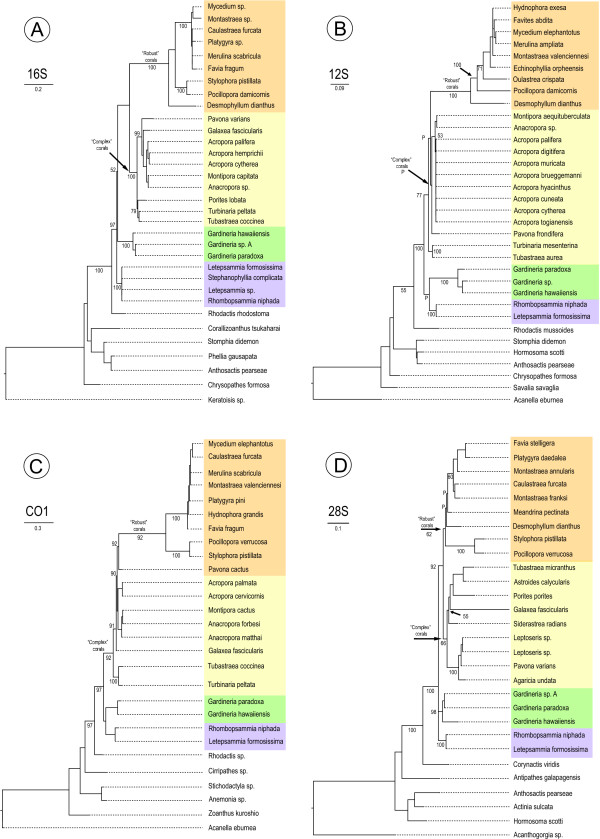
**Molecular phylograms based on 16S rDNA (A), 12S rDNA (B), COX1 (C) and 28S rDNA (D) sequences**. In each case, micrabaciid (highlighted purple) and gardineriid corals (highlighted green) are basal within the Scleractinia. Topologies were inferred by maximum likelihood, and numbers near branches leading to nodes represent the Bayesian posterior probabilities. Note that all but COX1 phylogeny recovered the early split between the Complexa and Robusta scleractinian clades.

Initial phylogenetic analyses were conducted on single gene sequences from a broad range of members of the anthozoan sub-Class (Hexacorallia/Zoantharia) to which corals belong. The application of maximum likelihood and Bayesian analyses to these datasets provided robust support for monophyly of the Scleractinia (Figure 1; see also Fukami et al. [16]), whereas paraphyly had been suggested in a previous study [17]. Moreover, as has recently been reported [15], the most deeply diverging scleractinian lineage was composed of representatives of the families Gardineriidae and Micrabaciidae, whose members are exclusively solitary and azooxanthellate.

**Figure 2:**
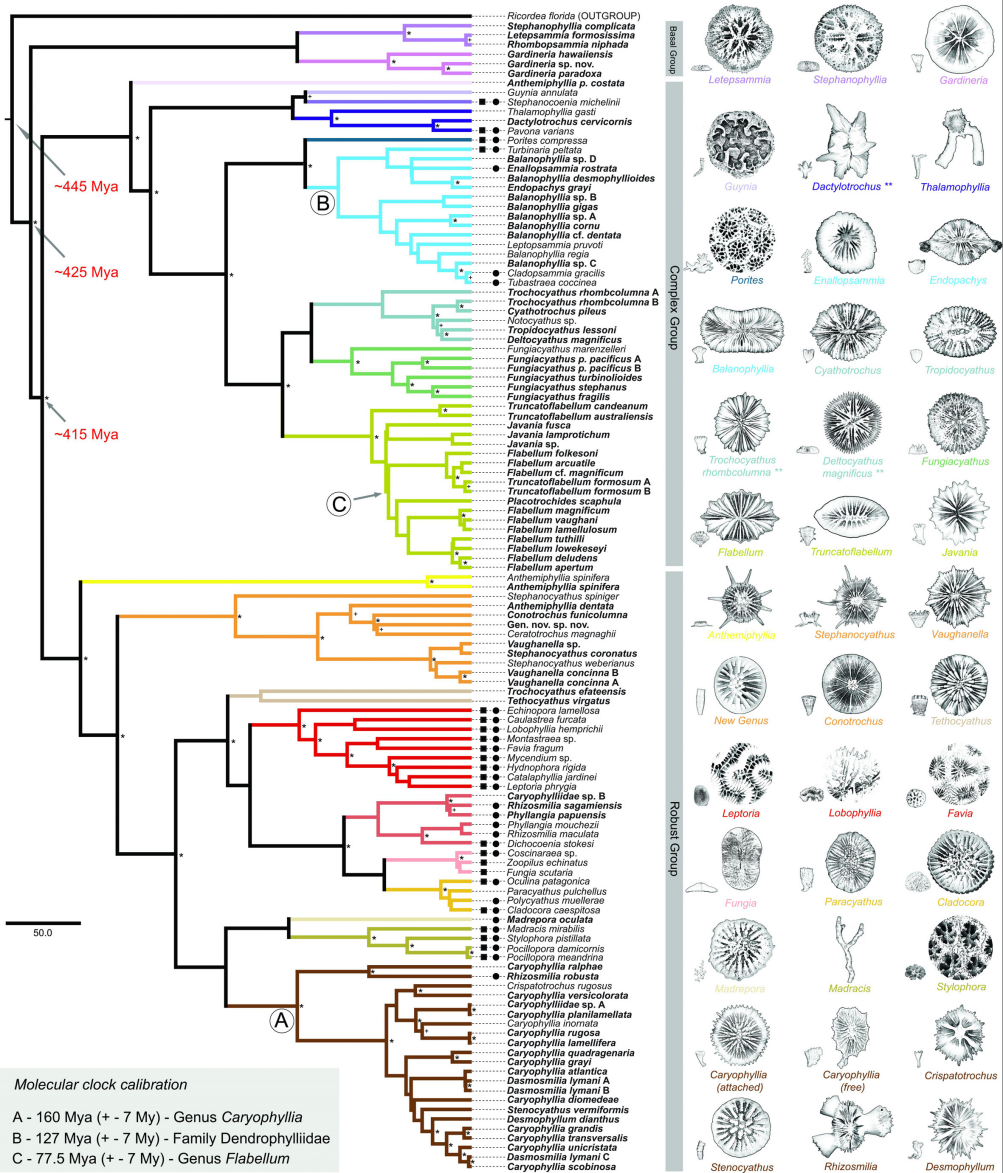
**Phylogeny of the Scleractinia based on Bayesian analysis of concatenated mitochondrial (16S rDNA) and nuclear (28S rDNA) data**. The tree shown is the majority rule consensus (BMC) cladogram based on sequence data for 121 scleractinian corals with the corallimorpharian *Ricordea florida *defined as outgroup. Representatives of the families Micrabaciidae and Gardineriidae form the basal clade within the Scleractinia, their divergence predating that of the Complexa and Robusta clades. To estimate divergence times for gardineriids/micrabaciids and other scleractinians, a relaxed molecular-clock (uncorrelated lognormal) Bayesian Markov chain Monte Carlo method was applied. The clock was calibrated using the earliest fossils that can be unambiguously assigned to extant clades and whose unique skeletal characters can be unequivocally recognized in fossil coralla (grey box identify each calibrated node and their respectively earliest fossil dates). Dates in red are discussed in the text. Asterisks (*) beside nodes indicate Maximum Likelihood (Chi-square and Bootstrap) and BMC (posterior probability) support greater than 0.95, 70, and 95 respectively, whereas a plus (+) indicates support higher than 0.80, 55, and 80 respectively. For each family/clade examined, the corresponding branches are colour coded. Black circles and/or black squares indicate those species that are colonial and/or zooxanthellate. Bold text indicates species for which sequence data was obtained in the present study. For the various scleractinian families included in the analyses, outlines of coralla for typical representatives (main - distal, and small - lateral/colony views) are shown to the right of the tree.

The second, more extensive, phase of phylogenetic analysis was carried out not only to clarify relationships within Scleractinia, but also to provide estimates of the timing of major divergences. For this purpose, 16S and 28S rDNA sequences were concatenated, but we avoided the creation of chimeric sequences (i.e. concatenation of sequences from different species) in these analyses. For the estimation of divergence times, the molecular-clock was calibrated using the oldest Mesozoic fossils that can be unequivocally assigned to extant genera/families, *Caryophyllia *for Caryophylliidae, *Flabellum *for Flabellidae, and *Palaeopsammia *for Dendrophylliidae (see Methods). As can be seen in Figure 2, these analyses imply that the basal clade comprising gardineriids and micrabaciids split with the major scleractinian lineage deep in the Paleozoic (ca. 425 Ma), significantly predating the Robusta/Complexa divergence, which our analyses place between the Silurian and Devonian (ca. 415 Ma) - more than 110 My earlier than previously thought [12]. In an attempt to test the accuracy of these divergence times, a second (Bayesian) relaxed molecular-clock analysis was performed on the coral dataset but with the inclusion of data for four homoscleromorph sponges as outgroups (data not shown). For this analysis, the same parameters were used, including the same calibration points, but forcing the root node - Homoscleromorpha/Eumetazoa split - to ca. 820 My (see Sperling et al. 2010, Table three [18]). Including the sponge data did not significantly affect the divergence time estimates for the main scleractinian nodes (Figure 2), indicating that these estimates are relatively robust, but did affect the estimate of the Corallimorpharia/Scleractinia divergence, placing it more than 50% deeper than previously estimated.

The discrepancy between the Complexa/Robusta divergence age estimated herein and those from previous studies may be due to a wider taxon sampling in the present study, and the quality [17] or absence of fossil calibration in the case of previous estimates [11,12]. For example, the first estimate of the timing of divergence between Complexa/Robusta [12] was based on comparison of 16S rDNA sequence divergence with that in Orders of holometabolous insects, making no allowance for the possibility of different rates of evolution. Additionally, recent divergence time analysis of the Holometabola origin is placed in the early Carboniferous (355 Ma), significantly older than in previous reconstructions [19].

The divergence of unambiguous scleractinians (gardineriids and micrabaciids) deep in the Paleozoic removes the temporal disconnect between Scleractinia and "scleractiniamorphs", the only substantial basis on which the two groups were previously distinguished. The known Paleozoic "scleractiniamorphs" were solitary or quasi-colonial (phaceloid), which, under the evolutionary scenario outlined below, is consistent with the idea that the ancestral scleractinian was solitary and azooxanthellate. Based on the clear similarity between "scleractiniamorph" skeletons and extant scleractinians, we consider that the Paleozoic "scleractiniamorphs" [7-10] should be reclassified as genuine scleractinians. Moreover, other (soft-bodied) hexacorallian fossils have been reported from as far back as the Cambrian [20], and the results presented here lend support to the idea that these might represent evolutionary precursors of the Scleractinia [21]. There are several possible explanations for the discontinuity of the Paleozoic record for Scleractinia. Paleozoic sediments containing corals may simply not yet have been found or are not preserved in the geological record. The only known lower Paleozoic scleractinian (genus *Kilbuchophyllia*) was recovered because shallow-water fossil-bearing deposits were transported to greater depth as the result of landslides [22]. This indicates that the currently known Paleozoic record might not be representative of the true diversity of the group at that time. Alternatively, skeletal formation in these early corals might have been an ephemeral trait [5,6], or skeleton-forming coral lineages went extinct. The same interpretative challenges apply to the evolutionary history of micrabaciids: their sudden appearance in the fossil record (Cretaceous) and lack of reliable ancestors among earlier scleractinian fauna suggest their emergence via skeletonization from an ancient "micrabaciid-gardineriid" skeleton-less hexacoral lineage, or points to huge gaps in the fossil record of deep-water scleractinians. Another important implication of the present analyses and those from the earlier COX1 analysis [15] is that modern shallow-water corals most likely had multiple independent origins from deep-water (azooxanthellate and solitary) ancestors (as has been hypothesized for another calcified cnidarian group, the Stylasteridae [23]), providing an explanation for the sudden appearance of the morphologically diverse Middle Triassic coral fauna.

Like their extant relatives, at least some Triassic Scleractinia hosted dinoflagellate symbionts - such associations conceivably evolved as a consequence of widespread oligotrophic conditions [24,25]. The explosive diversification of scleractinians in the Middle Triassic (ca. 240 Ma) coincides with a massive radiation of dinoflagellates [26], the former presumably being facilitated by the establishment of symbiosis.

**Figure 3:**
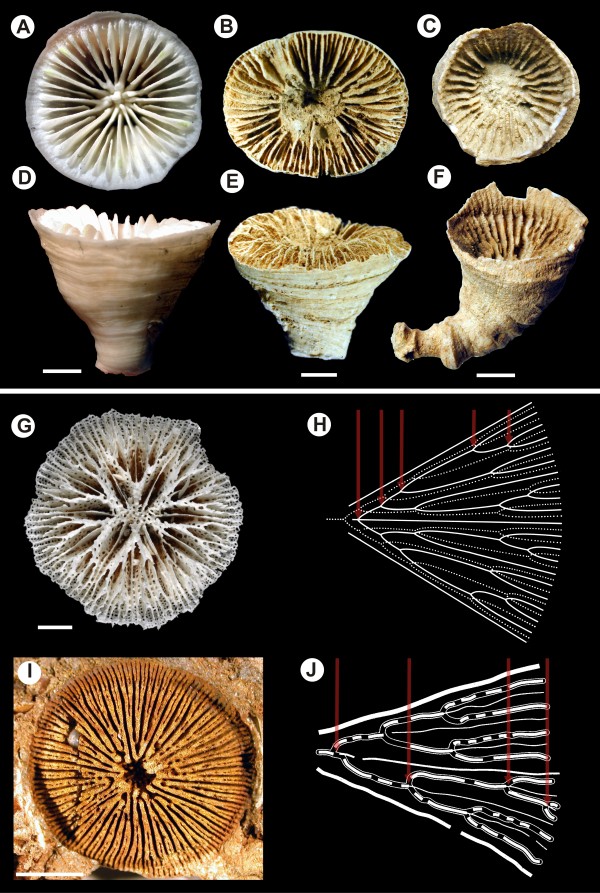
**Representatives of basal scleractinian clades (Gardineriidae, Micrabaciidae) vs some Mesozoic and Palaeozoic corals**. Overall morphological similarity between Recent *Gardineria *(A, D), some oldest known Mesozoic scleractinians (B, E; *Margarophyllia *sp., Triassic, ca. 230 Ma), and Palaeozoic rugosans (C, F; *Ptychophyllum *sp., Devonian, ca. 380 Ma), and morphological comparison between the skeleton of Recent micrabaciid *Letepsammia *(G, H), and mould of the Ordovician (ca. 460 Ma) *Kilbuchophyllia *(I, J). Despite the overall morphological similarity, resulting from occurrence of corrugated, entirely epithecal wall and relatively smooth septa, rugosans exhibit a different pattern of septal insertion than scleractinians (serial vs. cyclic, respectively), which most researchers consider the main argument of their independent origin. Calicular views (A-C); lateral views (D-F). A unique feature of modern micrabaciids is the multiple bifurcation of septa of the third order and straight and nonbifurcate septa of the first order: compare diagrammatic representation of one septal system in *Letepsammia *(H) and interpretation of the mould of *Kilbuchophyllia *(J); arrows indicate bifurcations of one branch of third order septa.

In terms of skeleton composition, septal insertion and overall anatomy (see Additional files 2, 3, 4, 5 and 6), micrabaciids and gardineriids are typical scleractinians [27], but these two families have unique features that distinguish them from each other and from all other extant Scleractinia [3,28]. Whilst shared morphological traits could reflect convergence, at least at a superficial level, the quite different gross skeletal architectures of gardineriids and micrabaciids are each strikingly reminiscent of more ancient coral and coral-like fossils. In gardineriids, the epithecal wall is the only wall of the corallum, which is an unusual feature among modern corals, but was prevalent among early Triassic scleractinians [6]; for example, *Margarophyllia *(Figure 3) or *Protoheterastraea *[3] from 230 Ma bear a striking resemblance to *Gardineria*. On the other hand, micrabaciids share a unique characteristic (bifurcating higher cycle septa) with kilbuchophyllid "scleractiniamorphs" (Figure 3) but, whereas the later had well-developed epithecal walls, this is not true for micrabaciids. Despite the basal position of the micrabaciid-gardineriid clade in scleractinian phylogeny, the first appearance of the micrabaciids in the fossil record is in the Cretaceous (Cenomanian, ca. 96 Ma) [29]. There are currently no earlier Triassic or Jurassic corals sharing septal organization and microstructural features with micrabaciids, so the ancestry of this family is again unclear [29]. The late appearance of micrabaciids in the fossil record is generally consistent with late (ca. 160 Ma, Middle Jurassic) divergence of micrabaciid and gardineriid lineages suggested by molecular phylogeny (Figure 2), but the lack of early Mesozoic micrabaciid-like fossils is puzzling.

## Conclusions

The analyses presented here support scleractinian monophyly and place the evolutionary origin of the Order deep in the Paleozoic, both of which are consistent with an independent origin from a soft-bodied ancestor but inconsistent with the rugosan ancestry hypothesis [1-3]. Although skeletal evidence is still lacking, the molecular data presented here bridge the gap in the fossil record between the Ordovician and Mesozoic Scleractinia. Although our results are robust and largely consistent with molecular-clock based analyses of other groups [18], the molecular markers used here may not be optimal for addressing deep-divergence events and should be verified using a range of additional markers.

The early origin of Scleractinia implied by our results has important implications for the debate about the fate of corals in times of global climate change, since they imply that the scleractinian lineage has persisted through several episodes of dramatic climate change during the last 450 My. Whilst on evolutionary time scales the Scleractinia may be less vulnerable than is sometimes assumed, the short-term survival of coral reefs as we know them is far less assured.

## Methods

### Material

The present study was based on the examination of 123 lots of deep-water azooxanthellate scleractinians collected from 87 stations from New Caledonia (French research expeditions Bathus 3, Bathus 4, Halipro 1, Norfolk 1 and Norfolk 2), and from Australia (Australian research expeditions SS 011997, SS 102005, SS 022007, and Tan0308). Additional specimens collected in Australian waters were provided by the Western Australian Museum (see Additional file 1).

### DNA preparation, amplification and sequence analyses

For large specimens, whole mesenteries were dissected out (with forceps) prior to extraction, whereas for smaller specimens an entire system (including skeleton) was extracted and immersed in the lysis buffer. Genomic DNA was extracted using the DNeasy Tissue Kit (QIAGEN). DNA concentrations were determined using a Nanodrop 1000 (Thermo Scientific) prior to Polymerase Chain Reaction (PCR) amplification under the following conditions:

(i) 16S rDNA - the primers developed by Le Goff-Vitry *et al. *[30] (LP16SF 5' -TTGACCGGTATGAATGGTGT and LP16SR 5' -TCCCCAGGGTAACTTTTATC) were used to amplify a fragment whose size varied between 280 and 420 bp. Reactions were carried out in a total volume of 50 μl, and contained 0.2 mM dNTPs, 1.5 mM MgCl2, 1 mM of each primer, 1.5 units of Taq polymerase (Fisher Biotec - Australia) and 125 ng of template. The PCR protocol used was: an initial denaturation step (95°C for 5 min), then 35 cycles of 30 s at 94°C, 30 s at 50°C, and 45 s at 72°C, followed by 10 min at 72°C.

(ii) COX1 - the universal primers developed by Folmer *et al. *[31] (LCO1 490 5' -GGTCAACAAATCATAAAGATATTGG and HCO2 198 5' -TAAACTTCAGGGTGACCAAAAAATCA) were used to amplify a fragment whose size varied between 690 and 710 bp. Reactions were carried out as described by Folmer *et al. *[31]: 95°C for 1 min, then 35 cycles of 30 s at 95°C, 30 s at 40°C, and 90 s at 72°C, followed by 10 min at 72°C.

(iii) 12S rDNA - the primers developed by Chen and Yu [32] (ANTMT12SF 5'-AGCCACACTTTCACTGAAACAAGG and ANTMT12SR 5'-GTTCCCYYWCYCTYACYATGTTACGAC) were used to amplify a fragment whose size varied between 800 and 920 bp. Reactions were carried out in 50 μl, with 0.2 mM dNTPs, 1.5 mM MgCl_2_, 1 mM of each primer, 1.5 units of Taq polymerase (Fisher Biotec - Australia), and 125 ng of template. PCR conditions were: 95°C for 4 min, followed by 4 cycles of 30 s at 94°C, 60 s at 50°C, 120 s at 72°C, and 30 cycles of 30 s at 94°C, 60 s at 55°C, 120 s at 72°C and then 4 min at 72°C.

(iv) 28S rDNA - the primers developed by Medina *et al. *[17] (28S.F63sq 5'-AATAAGCGGAGGAAAAGAAAC and 28S.R635sq 5'-GGTCCGTGTTTCAAGACGG) were used to amplify a fragment of approximately 750 bp. Reactions were carried out using the Advantage2 PCR kit (Clontech) with 100 ng of template, and following manufacturer's protocol. PCR conditions were: 95°C for 5 min, then 30 cycles of 30 s at 94°C, 60 s at 54°C, 90 s at 72°C, followed by 5 min at 72°C.

When amplification reactions based on Taq polymerase did not yield product, amplification was carried out using the Clontech Advantage-2 Kit (with the same template and primer concentrations, and under the same PCR protocol). PCR reactions were performed using a Bio-Rad DNA engine (Peltier Thermal Cycler). PCR products were purified using Mo-Bio Ultra Clean (PCR Clean Up) spin columns, and subjected to direct (Sanger) sequencing at Macrogen (South Korea).

Two different approaches were tested using sequences determined here and others retrieved from GenBank (see Additional file 1). The first approach included representatives of all hexacorallian orders but Ceriantharia and was intended to validate the Scleractinia monophyly. For this purpose, four single gene phylogenies all rooted with Octocorallia were constructed. The second approach, used herein for time divergence between scleractinian groups (Basal, Complex, and Robust groups), was based on concatenated sequences of the ribosomal genes16S rDNA and 28S rDNA, and included a broad range of scleractinian representatives. Alignments for both approaches were performed for each gene separately using ClustalW (EBI) and manually edited using JalView version 8.0 [33].

Alignments for the first approach were individually tested for substitution saturation [34] using DAMBE [35], which indicated little saturation for COX1 and 28S rDNA sequences (i.e. Iss. significantly lower than Iss.c), but higher levels of saturation for the 16S and 12S rDNAs (Iss. higher than Iss.c). Saturation related to mitochondrial ribosomal genes was induced by their respective fast evolving regions. This phenomenon was particularly evident because sequences from distant Anthozoa representatives were included in these alignments. To improve the phylogenetic signal, the most rapidly evolving regions were excluded from the alignment, resulting in a sharply decrease in saturation levels. The final alignments used in the first approach consisted of 298 positions for the16S rDNA, 599 positions for COX1, 631 positions for 12S rDNA, and 709 positions for the 28S rDNA. For each marker, appropriate models of nucleotide substitution were determined by the hierarchical likelihood ratio test implemented in MrModeltest [36]. Phylogenetic analyses were performed using PhyML [37] for maximum likelihood (ML) and MrBayes (version 3.1.2) [38] for Bayesian Inference (BI). The maximum likelihood analyses were performed under the GTR model with a non-parametric Shimodaira-Hasegawa-like procedure. For the Bayesian inference, two runs each of 10 million generations were calculated for each marker with topologies saved at each 1000 generations, with the average standard deviation of split frequencies between runs of each marker converging to or less than 0.01. The first quarter of the 10000 saved topologies were discarded as burnin, and the remaining used to calculate posterior probabilities (Figure 1).

The final alignment that based the second approach contained concatenated 16S rDNA and 28S rDNA sequences (without excluding the fast evolving regions) from 121 scleractinians and 1 corallimorpharian, totalling 1334 bp. This alignment was also tested for substitution saturation, which indicated good phylogenetic signal. ML phylogenetic analyses were performed as described above. However, instead of Shimodaira-Hasegawa-like statistical support, they were performed under the Chi-square and 100 bootstrap replicates.

To estimate divergence times for gardineriids/micrabaciids and other scleractinians, we applied a relaxed-clock (uncorrelated lognormal) Bayesian Markov chain Monte Carlo method as implemented in BEAST (version 1.4.8) [39]. This method allows nucleotide substitution rates to vary between lineages and incorporates phylogenetic uncertainty by sampling phylogenies and parameter estimates in proportion to their posterior probability. Additionally, Yule process was chosen as tree prior, and the prior distribution of divergence of each calibrated node was set as normal with standard deviation of 3.5. Hierarchical likelihood ratio tests led to the adoption of the General Time Reversible model with a proportion of invariant sites and gamma distributed rate heterogeneity (GTR+I+Γ) as the most appropriate evolutionary model for the molecular clock analyses. One run of 10 million generations was calculated with topologies and other parameters saved at each 1000 generations. A quarter of the 10000 saved topologies were discarded as burnin, and the remaining used to calculate posterior probabilities and node ages (Figure 1). Additionally, phylogenetic reconstruction from the same alignment was also calculated on MrBayes in two MCMC runs of 10 million generations each with topologies sampled every 1000 generations. Average standard deviation of split frequencies between runs was less than 0.01. The first quarter of the 10000 sampled topologies were discarded as burnin, and the remaining used to calculate posterior probabilities. The resulting topology was consistent with the one calculated using BEAST (data not shown).

For the calibration of the molecular clock, stringent constraints were applied based on fossils that can be unambiguously assigned to extant clades and whose unique skeletal characters can be unequivocally recognized in fossil coralla. Nodes used for the calibration were: (A) the appearance of *Caryophyllia *(ca. 160 Ma), based on the Late Jurassic (Oxfordian) species *C. simplex *and *C. suevica *[40,41]. Both species have well-developed "true" pali present in one crown before the penultimate cycle of septa, fascicular columella composed of several twisted laths and septothecal walls, characters which together occur only in fossil and extant representatives of this genus [42]; (B) The divergence of the Dendrophylliidae (ca. 127 Ma), corresponding to the first occurrence of solitary *Palaeopsammia *(Barremian) [43]. The first appearance of colonial dendrophylliids (*Blastozopsammia*) in the Albian (ca. 100 Ma) [44] is consistent with the earlier origin of solitary genera. Skeletal synapomorphies of dendrophylliids include the Pourtalès plan of septal arrangement and the presence of a synapticulothecate wall [45]; and (C) the origin of *Flabellum *(ca. 77.5 Ma), based on the earliest known record of the genus (*F. fresnoense*) from the Late Cretaceous (Coniacian; ?early Maastrichtian, based on foraminiferal assemblage) [46]. Unequivocal *Flabellum *fossils are also known from the Late Cretaceous (?Campanian, Maastrichtian) of Seymour Island (*F. anderssoni*) [47] and Late Cretaceous (Maastrichtian) of Western Australia (*Flabellum miriaensis*) [48]. *Flabellum *is clearly distinguishable based on the following unique combination of characters: the marginothecal wall is present throughout ontogeny, lack of pali/paliform lobes and scale-like microtexture of septa (sometimes preserved in the fossil record). Monophyly was enforced in the case of the dendrophylliids (calibration node B), but not for nodes A or C.

### Histological preparation

Ethanol preserved specimens were immersed in 20% (w/v) EDTA (pH 8) for two weeks at 4°C for decalcification. The resulting material was then dehydrated (70%, 80%, 2 × 95%, and 3 × 100% ethanol washes each of 40 min) and taken through three xylene washes (each of 40 min) prior to embedding in paraffin and serial tissue sectioning. Sections (5 μm) were stained using Harris's haematoxylin and eosin or Alcian Blue/PAS.

### Skeleton preparation and analysis

Preliminary selection of skeleton samples was performed using a Nikon SMZ800 stereoscopic zoom microscope. For standard SEM (Philips XL 20) measurements, polished and etched blocks of corals skeleton were used. Following published methods of preparation [49], the samples were polished with diamond powder, 1200 Grit and aluminium oxide (Buehler TOPOL) and then etched for 10 seconds in 0.1% formic acid. Trace element analyses were performed with the Cameca NanoSIMS N50 at the Muséum National d'Histoire Naturelle (Paris), following established procedures [50,51]. Briefly, septa were cut perpendicular to their growth direction, mounted in epoxy (Körapox^©^) and polished to a 0.25 μm finish using diamond paste. The samples were then gold-coated. Using a primary beam of O^-^, secondary ions of ^24^Mg^+^, ^44^Ca^+ ^and ^88^Sr^+ ^were sputtered from the sample surface and detected simultaneously (multicollection-mode) in electron-multipliers at a mass-resolving power of ~5000 (M/ΔM). At this mass-resolving power, the measured secondary ions are resolved from potential interferences. Data were obtained from a pre-sputtered surface as point analyses with the primary ions focused to a spot-size of ~3 micrometer and the primary beam stepped across the sample surface in steps of 20 micrometers. The measured ^24^Mg/^44^Ca and ^88^Sr/^44^Ca ratios were calibrated against analyses of carbonate standards of known composition (OKA-C) [52]. The chemical variations recorded in the coral skeletons are much larger than both the internal and external reproducibility of the standards, which were less than < 5% for Mg/Ca and < 3% for Sr/Ca. Briefly, the composition of skeletal trace elements of micrabaciids and gardineriids are consistent with other modern deep-sea corals [53], although the Sr/Ca ratios measured from *Letepsammia *are at the high end of the range: *Letepsammia *- Mg/Ca = 1-2 mmol/mol, Sr/Ca = 11.5-12.2 mmol/mol; *Stephanophyllia *- Mg/Ca = 2.5-3 mmol/mol, Sr/Ca = 10.3-11.2 mmol/mol; and *Gardineria *- Mg/Ca = 1.7-3.5 mmol/mol, Sr/Ca = 9.3-11.7 mmol/mol.

## Authors' contributions

The work presented here was carried out in collaboration between all authors. JS in cooperation with MVK defined the research theme, provided data on fossil corals, performed microstructural analyses of Recent skeleton samples and drafted the manuscript. MVK obtained the DNA sequences, performed the phylogenetic analyses, and helped to draft the manuscript. AM, MM performed additional analyses of the skeleton, discussed interpretation, and presentation, and helped to draft the manuscript. DM and SDC discussed interpretation and presentation as well as helping draft and edit the manuscript. All authors approved the final manuscript.

## Supplementary Material

Additional file 1 **Details for scleractinian specimens examined in the present study including GenBank accession data**. Species name and GenBank accession numbers for sequences determined in the present study are underlined. Whenever possible, multiple samples of each species from different collection stations were sequenced and the resulting consensus sequences used in the analyses. Click here for file

Additional file 2 **Anatomy of *Gardineria, Letepsammia *and other extant scleractinian corals**. The figure compares *Gardineria hawaiiensis *(A-E), *Letepsammia formosissima *(F-J), *Fungiacyathus margaretae *(K-O), and *Acropora millepora *(P-U) at the levels of skeleton macromorphology (first column), anatomy (second column) and histology (columns 3-4) (S-U, courtesy of Dr. Tracy Ainsworth). Color arrows indicate the following anatomical and histological details: black arrows, mouth/pharynx position on cross-sectioned polyps; gray arrows, septal position; pink arrows, spermaries, white arrows, calicoblastic ectoderm; yellow arrows, mesoglea; green arrows, mesogleal plates; red arrow, muscle fibers; dark blue arrows, zooxanthellae; light blue arrows, cnidae; orange arrows, mucocytes. All cross sections are stained with Alcian Blue/PAS or haematoxylin and eosin. Cnidae are shown on sections of tentacle acrospheres (E, J, O, U). *Fungiacyathus margaretae *and *Acropora millepora *were used as typical representatives of deep-water (azooxanthellate) and tropical shallow-water (zooxanthellate) Scleractinia respectively. Although the three deep-water species have significantly thicker mesoglea and mesogleal plates, and more abundant mucocytes than does the shallow water coral (*A. millepora*), *G. hawaiiensis *and *L. formosissima *are typical scleractinians in terms of all histological features examined. Click here for file

Additional file 3 **Initial ontogenetic stages in *Gardineria hawaiiensis *(A) and *Letepsammia formosissima *(B)**. The position of the six simultaneously inserted protosepta are indicated with white arrows. Thin section of the corallum base (A) and polished corallum base (B). Click here for file

Additional file 4 **Skeletons of modern, deep-water representatives of the Basal clade of Scleractinia: Gardineriidae (*Gardineria hawaiiensis*) and Micrabaciidae (*Letepsammia formosissima*)**. While gardineriids have very robust coralla (A, B), micrabaciids typically have a light, lace-like skeleton with perforated walls and septa (B, D, E). Such lightly calcified skeletons are common in corals living close to or below the carbonate compensation depth (4500-5000 m; see also Additional file 6). In addition, uniquely amongst extant corals, the thickening deposits of micrabaciids are composed of a meshwork of short and extremely thin (ca. 100-300 nm) fibers with variable crystallographic orientation (G, I). In the case of gardineriids, distinctively from most deep-water scleractinians, which display aragonite fibers in large bundles (e.g., *Desmophyllum*) or in complex patterns (e.g., *Flabellum*), septal microstructure typically forms smaller, vesicular units (F, H, see also Additional file 6). The cyclical insertion pattern of septa in gardineriids (A) and micrabaciids (B) is typical of Scleractinia. However, both taxa show several unique features that distinguish them from other modern corals and from one another. In *Gardineria *(C) the outer part of the skeleton consists of a thick epithecal wall, which is unique to modern corals but was common among the earliest solitary anthozoans. In contrast, the synapticular wall of micrabaciids is highly porous (D). Unique features of modern micrabaciids are the multiple bifurcations of septa of the third order, straight and non-bifurcate septa of the first order (B), and thickening deposits (TD) composed of irregular meshwork of short fibers organized into small bundles (G, I). In contrast, a central line of well-organized rapid accretion centers and radiating bundles of fibers, formed by sequentially addition of micrometer-sized growth layers characterize *Gardineria *septal microstructure (F, H). Distal (A, B), proximal (D), and lateral (C, E) views are shown. Transverse polished and etched sections (F-I) of septa of *G. hawaiiensis *(F, H) and *L. formosissima *(G, I) with Rapid Accretion Deposits (RAD) surrounded by bundles of Thickening Deposits (TD). Scale bars 10 mm (A-E), and 20 μm (F-I). Click here for file

Additional file 5 **Microstructural features of *Letepsammia *(Micrabaciidae), *Gardineria *(Gardineriidae) and other Recent scleractinian corals**. The SEM micrographs shown are of etched polished surfaces of septa. In addition to differences in the distribution of Rapid Accretion Deposits (RAD), major differences can also be seen in the arrangement of the thickening deposits (TD). In *Letepsammia formosissima *(A), the TDs are composed of an irregular meshwork of fiber bundles oriented sub-parallel to the surface, whereas in *Gardineria hawaiiensis *(B), bundles of fibers (TD) form smaller, vesicular units. In *Desmophyllum dianthus *(C), *Caryophyllia cyathus *(D) and *Favia stelligera *(G), the TDs consist of bundles of fibers running perpendicular to the skeletal surface (in the case of the zooxanthellate coral *Favia*, these display high regularity, corresponding to daily growth increments). The TDs in *Flabellum *(E), *Galaxea *(F), and *Acropora *(H) show micro-laminar organization corresponding to the scale-like micro-texture of their skeleton surfaces. Click here for file

Additional file 6 **Abyssal scleractinians**. Of known scleractinians, representatives of *Leptopenus *(A, B) and *Fungiacyathus *(C, D) occur at the greatest depths (reaching depths > 5000), consequently developing fragile and thin skeletons of low density. The upper two images (A, B) are of a formaldehyde preserved specimen of *Leptopenus*, the bulk of the animal being composed of soft tissue (brown); the delicate skeleton (white) is deeply embedded within the polyp tissue. The two lower images (C, D) show the extremely thin, parchment-like skeleton of *Fungiacyathus*. Proximal views. Click here for file
