# Supplementary material for: The ancient evolutionary origins of Scleractinia revealed by azooxanthellate corals
Source: BMC Evol Biol. 2011 Oct 28;11:316. doi: 10.1186/1471-2148-11-316 (PMC3224782; doi:10.1186/1471-2148-11-316)
Supplement: Additional file 1 — Details for scleractinian specimens examined in the present study including GenBank accession data. Species name and GenBank accession numbers for sequences determined in the present study are underlined. Whenever possible, multiple samples of each species from different collection stations were sequenced and the resulting consensus sequences used in the analyses. [file 1471-2148-11-316-S1.DOC]

| **Taxonomy**  **Family /** Species | **Expedition** | | **Station** | | **Skeletal voucher location** | | **Station details** | | | | | | **Specimens sequenced and**  **GenBank Accession numbers** | | | | | | |
| --- | --- | --- | --- | --- | --- | --- | --- | --- | --- | --- | --- | --- | --- | --- | --- | --- | --- | --- | --- |
|  |  |  |  |  |  |  | **Lat (S)** | | **Long (E)** | | **Depth (m)** | | **12S rRNA** | | **16S rRNA** | | **COX1** | | **28S rRNA** |
| **SCLERACTINIA** | | | | | | | | | | | | | | | | | | | |
| **Acroporiidae** | | | | | | | | | | | | | | | | | | | |
| *Acropora brueggemanni* | - | | - | | - | | - | | - | | - | | AF333048 | | - | | - | | - |
| *Acropora cervicornis* | - | | - | | - | | - | | - | | - | | - | | - | | AY451340 | | - |
| *Acropora cuneata* | - | | - | | - | | - | | - | | - | | AF333049 | | - | | - | | - |
| *Acropora cytherea* | - | | - | | - | | - | | - | | - | | AF333054 | | L75995 | | - | | - |
| *Acropora digitifera* | - | | - | | - | | - | | - | | - | | AF333051 | | - | | - | | - |
| *Acropora hyacinthus* | - | | - | | - | | - | | - | | - | | AF333053 | | - | | - | | - |
| *Acropora hemprichii* | - | | - | | - | | - | | - | | - | | - | | AF550359 | | - | | - |
| *Acropora muricata* | - | | - | | - | | - | | - | | - | | AF333052 | | - | | - | | - |
| *Acropora palifera* | - | | - | | - | | - | | - | | - | | AF333047 | | AF265593 | | - | | - |
| *Acropora palmate* | - | | - | | - | | - | | - | | - | | - | | - | | AB441246 | | - |
| *Acropora togianensis* | - | | - | | - | | - | | - | | - | | AF333050 | | - | | - | | - |
| *Anacropora forbesi* | - | | - | | - | | - | | - | | - | | - | | - | | AB441251 | | - |
| *Anacropora matthai* | - | | - | | - | | - | | - | | - | | - | | - | | AB441250 | | - |
| *Anacropora* sp. | - | | - | | - | | - | | - | | - | | AF333046 | | L75992 | | - | | - |
| *Montipora aequituberculata* | - | | - | | - | | - | | - | | - | | AF333045 | | - | | - | | - |
| *Montipora cactus* | - | | - | | - | | - | | - | | - | | - | | - | | AB441252 | | - |
| *Montipora capitata* | - | | - | | - | | - | | - | | - | | - | | L76015 | | - | | - |
| **Agariciidae** | | | | | | | | | | | | | | | | | | | |
| *Agaricia humilis* | - | | - | | - | | - | | - | | - | | - | | - | | AB441219 | | - |
| *Agaricia undata* | - | | - | | - | | - | | - | | - | | - | | - | | - | | EU262789 |
| *Leptoseris cucullata* | - | | - | | - | | - | | - | | - | | - | | - | | AB441220 | | - |
| *Leptoseris* sp. | - | | - | | - | | - | | - | | - | | - | | - | | AY451373 | | EU262806 |
| *Pavona frondifera* | - | | - | | - | | - | | - | | - | | AF333055 | | - | | - | | - |
| *Pavona varians* | - | | - | | - | | - | | - | | - | | - | | L76016 | | - | | EU262847 |
| **Anthemiphylliidae** | | | | | | | | | | | | | | | | | | | |
| *Anthemiphyllia patera costata* | Norfolk 2 | | DW2080 | | JCU | | 25º20’ | | 168º19’ | | 764-816 | | - | | - | | - | | HQ439609 |
| *Anthemiphyllia patera costata* | Norfolk 2 | | DW2080 | | JCU | | 25º20’ | | 168º19’ | | 764-816 | | - | | - | | - | | - |
| *Anthemiphyllia patera costata* | Norfolk 2 | | DW2080 | | JCU | | 25º20’ | | 168º19’ | | 764-816 | | - | | HQ439684 | | - | | - |
| *Anthemiphyllia spinifera* | - | | - | | - | | - | | - | | - | | - | | AF265596 | | - | | EU262852 |
| *Anthemiphyllia spinifera* | Norfolk 2 | | DW2142 | | JCU | | 23º01’ | | 168º17’ | | 550 | | - | | HQ439685 | | - | | HQ439610 |
| *Anthemiphyllia dentate* | SS102005 | | 006-049 | | CSIRO | | 31º36’31’’ | | 114º58’19’’ | | 329-370 | | - | | HQ439686 | | - | | - |
| *Anthemiphyllia dentate* | Norfolk 2 | | DW2147 | | JCU | | 22º50’ | | 167º16’ | | 496 | | - | | - | | - | | HQ439611 |
| **Astrocoeniidae** | | | | | | | | | | | | | | | | | | | |
| *Stephanocoenia michelinii* | - | | - | | - | | - | | - | | - | | - | | AF265581 | | - | | EU262865 |
| **Caryophylliidae** | | | | | | | | | | | | | | | | | | | |
| *Caryophyllia atlantica* | SS022007 | 050-050 | | CSIRO | | 44º12’4’’ | | 146º11’56’’ | | 1050-1230 | | - | | FJ788113 | | - | | HQ439612 | |
| *Caryophyllia diomedeae* | Norfolk 2 | DW2106 | | JCU | | 23º54’ | | 167º42’ | | 685-757 | | - | | FJ788114 | | - | | - | |
| *Caryophyllia diomedeae* | SS022007 | 066-014 | | CSIRO | | 43º51’40’’ | | 150º25’42’’ | | 800-1000 | | - | | FJ788115 | | - | | HQ439613 | |
| *Caryophyllia grandis* | - | Z13094 | | WAM | | - | | - | | - | | - | | FJ788117 | | - | | HQ439614 | |
| *Caryophyllia grandis* | SS102005 | 171-033 | | CSIRO | | 21º00’22’’ | | 114º22’51’’ | | 399-411 | | - | | FJ788118 | | - | | - | |
| *Caryophyllia grayi* | SS102005 | 1??-47 | | CSIRO | | - | | - | | - | | - | | FJ788119 | | - | | HQ439615 | |
| *Caryophyllia inornata* | - | - | | - | | - | | - | | - | | - | | AF265599 | | - | | EU262777 | |
| *Caryophyllia lamellifera* | Norfanz0308 | 24 | | CSIRO | | 28º32’38’’ | | 167º24’37’’ | | 111-115 | | - | | FJ788120 | | HM018616 | | HQ439616 | |
| *Caryophyllia planilamellata* | SS102005 | 20-008 | | CSIRO | | 35º22’54’’ | | 117º12’10’’ | | 419-460 | | - | | FJ788121 | | - | | HQ439617 | |
| *Caryophyllia planilamellata* | SS102005 | 040-13 | | CSIRO | | 35º13’38’’ | | 118º35’38’’ | | 398-554 | | - | | FJ788122 | | - | | - | |
| *Caryophyllia quadragenaria* | Norfolk 2 | DW2159 | | JCU | | 22º41’ | | 167º12’ | | 300-305 | | - | | HQ439687 | | - | | HQ439618 | |
| *Caryophyllia ralphae* | Norfolk 2 | DW2136 | | JCU | | 23º01’ | | 168º23’ | | 402-410 | | - | | HQ439688 | | - | | HQ439619 | |
| *Caryophyllia rugosa* | Norfanz0308 | 57 | | CSIRO | | 29º07’50’’ | | 159º00’15’’ | | 300 | | - | | FJ788123 | | HM018618 | | HQ439620 | |
| *Caryophyllia scobinosa* | SS102005 | 150-08 | | CSIRO | | 22º00’13’’ | | 113º40’44’’ | | 983-1010 | | - | | FJ788124 | | - | | HQ439621 | |
| *Caryophyllia smithii* | - | - | | - | | - | | - | | - | | - | | - | | - | | AF549216 | |
| *Caryophyllia transversalis* | - | Z13144 | | WAM | | - | | - | | - | | - | | FJ788126 | | - | | HQ439622 | |
| *Caryophyllia transversalis* |  | Z13144 | | WAM | |  | |  | |  | |  | | FJ788125 | |  | |  | |
| *Caryophyllia unicristata* | - | Z13142 | | WAM | | - | | - | | - | | - | | FJ788127 | | - | | - | |
| *Caryophyllia unicristata* | SS102005 | 171-02 | | CSIRO | | 21º00’22’’ | | 114º22’51’’ | | 399-411 | | - | | FJ788128 | | - | | HQ439623 | |
| *Caryophyllia unicristata* | SS102005 | 172-08 | | CSIRO | | 21º00’24’’ | | 114º22’51’’ | | 399-408 | | - | | FJ788129 | | - | | - | |
| *Caryophyllia versicolorata* | Norfolk 2 | DW2035 | | JCU | | 23º40’ | | 167º40’ | | 515-540 | | - | | HQ439689 | | - | | - | |
| *Caryophyllia versicolorata* | Norfolk 2 | DW2036 | | JCU | | 23º38’ | | 167º39’ | | 571-610 | | - | | HQ439690 | | - | | HQ439624 | |
| Caryophylliidae sp. A | SS102005 | 017-034 | | CSIRO | | 35º04’10’’ | | 115º20’09’’ | | 378-379 | | - | | HQ439691 | | - | | HQ439625 | |
| Caryophylliidae sp. B | SS102005 | 096 | | CSIRO | | 27º48’28’’ | | 113º17’49’’ | | 112-123 | | - | | HQ439692 | | - | | HQ439626 | |
| Caryophylliidae Gen. nov. sp. Nov | Bathus 4 | DW923 | | JCU | | 18º51.51’ | | 163º24.17’ | | 470-502 | | - | | - | | - | | HQ439627 | |
| Caryophylliidae Gen. nov. sp. Nov | Norfolk 2 | DW2035 | | JCU | | 23º40’ | | 167º40’ | | 515-540 | | - | | HQ439693 | | - | | - | |
| Caryophylliidae Gen. nov. sp. Nov | Norfolk 2 | DW2035 | | JCU | | 23º40’ | | 167º40’ | | 515-540 | | - | | HQ439694 | |  | | HQ439628 | |
| *Ceratotrochus magnaghii* | - | - | | - | | - | | - | | - | | - | | AF265597 | | - | | EU262879 | |
| *Cladocora caespitose* | - | - | | - | | - | | - | | - | | - | | AF265612 | | - | | EU262802 | |
| *Conotrochus funicolumna* | SS022007 | 006-056 | | CSIRO | | 43º59’29’’ | | 147º32’46’’ | | 370-410 | | - | | HQ439695 | | - | | HQ439629 | |
| *Conotrochus funicolumna* | Norfolk 2 | DW2035 | | JCU | | 23º40’ | | 167º40’ | | 515-540 | | - | | HQ439696 | | - | | - | |
| *Crispatotrochus rugosus* | - | - | | - | | - | | - | | - | | - | | AF265600 | | - | | EU262860 | |
| *Dactylotrochus cervicornis* | Norfolk 2 | DW2135 | | JCU | | 23º02’ | | 168º21’ | | 295-330 | | - | | HQ439697 | | - | | HQ439630 | |
| *Dasmosmilia lymani A* | SS102005 | 149-019 | | CSIRO | | 22º03’34’’ | | 113º43’44’’ | | 658-754 | | - | | HQ439766 | | - | | HQ439631 | |
| *Dasmosmilia lymani B* | SS102005 | 25-014 | | CSIRO | | 35º21’52’’ | | 118º18’25’’ | | 398-407 | | - | | HQ439767 | | - | | HQ439632 | |
| *Dasmosmilia lymani C* | SS022007 | 008-012 | | CSIRO | | 31º40’50’’ | | 114º50’45’’ | | 669-683 | | - | | HQ439768 | | - | | HQ439633 | |
| *Deltocyathus magnificus* | SS102005 | 128-07 | | CSIRO | | 23º59’06’’ | | 112º32’42’’ | | 398-402 | | - | | HQ439769 | | - | | HQ439634 | |
| *Deltocyathus magnificus* | SS102005 | 016-012 | | CSIRO | | 34º00’39’’ | | 114º26’34’’ | | 467-490 | | - | | HQ439770 | | - | | - | |
| *Deltocyathus magnificus* | SS102005 | 68-011 | | CSIRO | | 31º59’32’’ | | 115º10’58’’ | | 478-508 | | - | | HQ439771 | | - | | - | |
| *Deltocyathus magnificus* | SS102005 | 013 | | CSIRO | | 33º00’30’’ | | 114º34’15’’ | | 414-421 | | - | | HQ439772 | | - | | - | |
| *Desmophyllum dianthus* | SS011997 | 022 | | CSIRO | | 44º21’ | | 147º15’ | | ? | | - | | GQ868694 | | - | | - | |
| *Desmophyllum dianthus* | Norfolk 2 | DW2132 | | JCU | | 23º17’ | | 168º14’ | | 405-455 | | GQ868667 | | GQ868690 | | - | | - | |
| *Desmophyllum dianthus* | Norfolk 2 | DW2142 | | JCU | | 23º01’ | | 168º17’ | | 550 | | GQ868666 | | - | | - | | GQ868676 | |
| *Paracyathus pulchellus* | - | - | | - | | - | | - | | - | | - | | AF265603 | | - | | EU262820 | |
| *Phyllangia mouchezii* | - | - | | - | | - | | - | | - | | - | | AF265605 | | - | | EU262798 | |
| *Phyllangia papuensis* | SS102005 | 091-44 | | CSIRO | | 28º59’19’’ | | 113º47’02’’ | | 180-183 | | - | | HQ439773 | | - | | HQ439669 | |
| *Polycyathus muellerae* | - | - | | - | | - | | - | | - | | - | | AF265606 | | - | | EU262790 | |
| *Rhizosmilia maculate* | - | - | | - | | - | | - | | - | | - | | AF265602 | | - | | EU262796 | |
| *Rhizosmilia robusta* | Norfolk 2 | DW2135 | | JCU | | 23º02’ | | 168º21’ | | 295-330 | | - | | HQ439698 | | - | | HQ439635 | |
| *Rhizosmilia sagamiensis* | Norfolk 2 | DW2135 | | JCU | | 23º02’ | | 168º21’ | | 295-330 | | - | | HQ439699 | | - | | HQ439636 | |
| *Stephanocyathus coronatus* | Halipro 1 | CP854 | | JCU | | 22º05.03’ | | 166º38.34’ | | 650-780 | | - | | HQ439700 | | - | | - | |
| *Stephanocyathus coronatus* | Halipro 1 | CP854 | | JCU | | 22º05.03’ | | 166º38.34’ | | 650-780 | | - | | HQ439701 | | - | | HQ439637 | |
| *Stephanocyathus spiniger* | Norfolk 2 | DW2137 | | JCU | | 23º01’ | | 168º23’ | | 547-560 | | - | | - | | - | | HQ439638 | |
| *Stephanocyathus spiniger* | - | - | | - | | - | | - | | - | | - | | HM015359 | | - | | - | |
| *Stephanocyathus weberianus* | - | - | | - | | - | | - | | - | | - | | AF265594 | | - | | EU262795 | |
| *Tethocyathus virgatus* | Norfolk 2 | DW2057 | | JCU | | 24º40’ | | 168º39’ | | 555-565 | | - | | FJ788131 | | - | | HQ439639 | |
| *Tethocyathus virgatus* | Norfolk 2 | DW2084 | | JCU | | 24º52’ | | 168º22’ | | 586-730 | | - | | HQ439702 | | - | | - | |
| *Thalamophyllia gasti* | - | - | | - | | - | | - | | - | | - | | AF265590 | | - | | EU262788 | |
| *Trochocyathus efateensis* | Norfolk 2 | DW2132 | | JCU | | 23º17’ | | 168º14’ | | 405-455 | | - | | FJ788132 | | - | | HQ439640 | |
| *Trochocyathus rhombcolumna A* | Norfolk 2 | DW2157 | | JCU | | 22º56’ | | 167º19’ | | 553-575 | | - | | HQ439703 | | - | | HQ439641 | |
| *Trochocyathus rhombcolumna B* | Norfolk 2 | DW2132 | | JCU | | 23º17’ | | 168º14’ | | 405-455 | | - | | HQ439704 | | - | | HQ439642 | |
| *Vaughanella concinna* | Norfolk 2 | DW2075 | | JCU | | 25º23’ | | 168º20’ | | 650-1000 | | - | | HQ439705 | | - | | HQ439643 | |
| *Vaughanella concinna* | Norfolk 2 | DW2075 | | JCU | | 25º23’ | | 168º20’ | | 650-1000 | | - | | HQ439706 | | - | | - | |
| *Vaughanella concinna* | Norfolk 2 | DW2106 | | JCU | | 23º54’ | | 167º42’ | | 685-757 | | - | | HQ439707 | | - | | HQ439644 | |
| *Vaughanella* sp. 1 | Norfolk 2 | DW2080 | | JCU | | 25º20’ | | 168º19’ | | 764-816 | | - | | HQ439708 | | - | | HQ439645 | |
| **Dendrophylliidae** | | | | | | | | | | | | | | | | | | | |
| *Astroides calycularis* | - | | - | | - | | - | | - | | - | | - | | - | | - | | AF549248 |
| *Balanophyllia* cf. *dentate* | SS102005 | | 096 | | CSIRO | | 27º48’28’’ | | 113º17’49’’ | | 112-123 | | - | | HQ439709 | | - | | HQ439646 |
| *Balanophyllia cornu* | SS102005 | | 140-16 | | CSIRO | | 22º37’03’’ | | 113º29’02’’ | | 355-382 | | - | | HQ439710 | | - | | HQ439647 |
| *Balanophyllia desmophyllioides* | SS102005 | | 140-12 | | CSIRO | | 22º37’03’’ | | 113º29’02’’ | | 355-382 | | - | | HQ439711 | | - | | - |
| *Balanophyllia desmophyllioides* | Norfolk 2 | | DW2119 | | JCU | | 23º23’ | | 168º02’ | | 300 | | - | | - | | - | | HQ439648 |
| *Balanophyllia gigas* | Norfolk 2 | | CP2141 | | JCU | | 23º01’ | | 168º20’ | | 92-100 | | - | | HQ439712 | | - | | HQ439649 |
| *Balanophyllia regia* | - | | - | | - | | - | | - | | - | | - | | AF265587 | | - | | EU262813 |
| *Balanophyllia* sp. 1 | Norfolk 2 | | DW2057 | | JCU | | 24º40’ | | 168º39’ | | 555-565 | | - | | HQ439713 | | - | | HQ439650 |
| *Balanophyllia* sp. 2 | SS102005 | | 115-48 | | CSIRO | | 25º55’46’’ | | 112º40’48’’ | | 120 | | - | | HQ439714 | | - | | HQ439651 |
| *Balanophyllia* sp. 3 | SS102005 | | 110-044 | | CSIRO | | 27º03’07’’ | | 113º04’51’’ | | 106 | | - | | HQ439715 | | - | | HQ439652 |
| *Balanophyllia* sp. 4 | Norfolk 2 | | DW2132 | | JCU | | 23º17’ | | 168º14’ | | 405-455 | | - | | HQ439716 | | - | | HQ439653 |
| *Cladopsammia gracilis* | - | | - | | - | | - | | - | | - | | - | | AF265588 | | - | | EU262774 |
| *Enallopsammia rostrata* | Norfolk 2 | | DW2075 | | JCU | | 25º23’ | | 168º20’ | | 650-1000 | | - | | HQ439718 | | - | | - |
| *Enallopsammia rostrata* | Norfolk 2 | | DW2075 | | JCU | | 25º23’ | | 168º20’ | | 650-1000 | | - | | - | | - | | HQ439654 |
| *Enallopsammia rostrata* | Norfolk 2 | | CP2121 | | JCU | | 23º23’ | | 168º00’ | | 486-514 | | - | | HQ439717 | | - | | - |
| *Endopachys grayi* | Norfolk 2 | | DW2159 | | JCU | | 22º41’ | | 167º12’ | | 300-305 | | - | | HQ439719 | | - | | HQ439655 |
| *Leptopsammia pruvoti* | - | | - | | - | | - | | - | | - | | - | | AF265579 | | - | | EU262851 |
| *Tubastraea aurea* | - | | - | | - | | - | | - | | - | | AF333064 | | - | | - | | - |
| *Tubastraea coccinea* | - | | - | | - | | - | | - | | - | | - | | L76022 | | AB441235 | | EU262864 |
| *Tubastraea micranthus* | - | | - | | - | | - | | - | | - | | - | | - | | - | | AF549219 |
| *Turbinaria mesenterina* | - | | - | | - | | - | | - | | - | | AF333063 | | - | | - | | - |
| *Turbinaria peltata* | - | | - | | - | | - | | - | | - | | - | | L76023 | | AB441240 | | EU262799 |
| **Euphylliidae** | | | | | | | | | | | | | | | | | | | |
| *Catalaphyllia jardinei* | - | | - | | - | | - | | - | | - | | - | | L76000 | | - | | EU262874 |
| **Faviidae** | | | | | | | | | | | | | | | | | | | |
| *Caulastrea furcata* | - | | - | | - | | - | | - | | - | | - | | L75997 | | AB117274 | | AF549224 |
| *Echinopora lamellose* | - | | - | | - | | - | | - | | - | | - | | L76003 | | - | | EU262773 |
| *Favia fragum* | - | | - | | - | | - | | - | | - | | - | | FFU40295 | | AY451351 | | EU262856 |
| *Favia stelligera* | - | | - | | - | | - | | - | | - | | - | | - | | - | | AF549223 |
| *Favites abdita* | - | | - | | - | | - | | - | | - | | AF333060 | | - | | - | | - |
| *Leptoria phrygia* | - | | - | | - | | - | | - | | - | | - | | L76011 | | - | | AF549228 |
| *Montastraea annularis* | - | | - | | - | | - | | - | | - | | - | | - | | - | | AF549229 |
| *Montastraea curta* | - | | - | | - | | - | | - | | - | | - | | - | | - | | AF549230 |
| *Montastraea franksi* | - | | - | | - | | - | | - | | - | | - | | - | | AP008976 | | - |
| *Montastraea* sp. | - | | - | | - | | - | | - | | - | | - | | AF265610 | | - | | EU262794 |
| *Montastraea valenciennesi* | - | | - | | - | | - | | - | | - | | AF333061 | | - | | - | | - |
| *Oulastrea crispate* | - | | - | | - | | - | | - | | - | | AF333062 | | - | | - | | - |
| *Platygyra daedalea* | - | | - | | - | | - | | - | | - | | - | | - | | - | | AF549231 |
| *Platygyra pini* | - | | - | | - | | - | | - | | - | | - | | - | | EU371722 | | - |
| *Platygyra* sp. | - | | - | | - | | - | | - | | - | | - | | AF265611 | | - | | - |
| **Flabellidae** | | | | | | | | | | | | | | | | | | | |
| *Flabellum apertum* | SS022007 | | 050-024 | | CSIRO | | 44º12’04’’ | | 146º11’56’’ | | 1050-1230 | | - | | HQ439720 | | - | | HQ439656 |
| *Flabellum arcuatile* | Norfolk 2 | | DW2132 | | JCU | | 23º17’ | | 168º14’ | | 405-455 | | - | | HQ439721 | | - | | HQ439657 |
| *Flabellum arcuatile* | Norfolk 2 | | DW2132 | | JCU | | 23º17’ | | 168º14’ | | 405-455 | | - | | HQ439722 | | - | | - |
| *Flabellum* cf. *magnificum* | SS102005 | | 172-051 | | CSIRO | | 21º00’24’’ | | 114º22’51’’ | | 399-408 | | - | | HQ439723 | | - | | HQ439658 |
| *Flabellum deludens* | SS022007 | | 008-009 | | CSIRO | | 31º40’50’’ | | 114º50’45’’ | | 669-683 | | - | | HQ439724 | | - | | HQ439659 |
| *Flabellum folkesoni* | - | | Z13161 | | WAM | | - | | - | | - | | - | | HQ439725 | | - | | - |
| *Flabellum folkesoni* | SS102005 | | 171-033 | | CSIRO | | 21º00’22’’ | | 114º22’51’’ | | 399-411 | | - | | - | | - | | HQ439660 |
| *Flabellum lamellulosum* | SS102005 | | 171-033 | | CSIRO | | 21º00’22’’ | | 114º22’51’’ | | 399-411 | | - | | HQ439726 | | - | | HQ439661 |
| *Flabellum lamellulosum* | SS102005 | | 122-017 | | CSIRO | | 24º33’7’’ | | 112º15’07’’ | | 396-404 | | - | | HQ439727 | | - | | - |
| *Flabellum lowekeseyi* | SS022007 | | 041-27 | | CSIRO | | 44º03’56’’ | | 146º14’02’’ | | 800-880 | | - | | HQ439728 | | - | | - |
| *Flabellum lowekeseyi* | SS022007 | | 059-036 | | CSIRO | | 44º04’09’’ | | 147º25’24’’ | | 810-1020 | | - | | HQ439729 | | - | | HQ439662 |
| *Flabellum lowekeseyi* | SS022007 | | 008-009 | | CSIRO | | 44º01’50’’ | | 147º34’46’’ | | 830-1030 | | - | | HQ439730 | | - | | - |
| *Flabellum lowekeseyi* | SS022007 | | 008-009 | | CSIRO | | 44º01’50’’ | | 147º34’46’’ | | 830-1030 | | - | | HQ439731 | | - | | - |
| *Flabellum magnificum* | - | | Z13223 | | WAM | | - | | - | | - | | - | | HQ439732 | | - | | HQ439663 |
| *Flabellum tuthilli* | SS102005 | | 034-056 | | CSIRO | | 35º12’48’’ | | 118º39’03’’ | | 408-431 | | - | | HQ439733 | | - | | HQ439664 |
| *Flabellum tuthilli* | SS102005 | | 033-008 | | CSIRO | | 35º11’26’’ | | 118º38’41’’ | | 147-157 | | - | | HQ439734 | | HM018643 | | - |
| *Flabellum vaughani* | SS102005 | | 130-009 | | CSIRO | | 23º59’11’’ | | 112º32’02’’ | | 411 | | - | | HQ439735 | | - | | HQ439665 |
| *Flabellum vaughani* | ? | | ? | | CSIRO | | - | | - | | - | | - | | HQ439736 | | - | | - |
| *Javania fusca* | Norfolk 2 | | DW2064 | | JCU | | 25º17’ | | 168º56’ | | 609-691 | | - | | HQ439737 | | - | | - |
| *Javania fusca* | Norfolk 2 | | DW2064 | | JCU | | 25º17’ | | 168º56’ | | 609-691 | | - | | HQ439738 | | - | | HQ439666 |
| *Javania fusca* | Norfolk 2 | | DW2106 | | JCU | | 23º54’ | | 167º42’ | | 685-757 | | - | | HQ439739 | | - | | - |
| *Javania lamprotichum* | SS022007 | | 059-036 | | CSIRO | | 44º04’09’’ | | 147º25’24’’ | | 810-1020 | | - | | HQ439740 | | - | | - |
| *Javania lamprotichum* | SS022007 | | 016-015 | | CSIRO | | 44º19’32’’ | | 147º10’30’’ | | 1100-1160 | | - | | HQ439741 | | - | | HQ439667 |
| *Javania lamprotichum* | SS022007 | | 041-027 | | CSIRO | | 44º03’56’’ | | 146º14’02’’ | | 800-880 | | - | | HQ439742 | | - | | - |
| *Javania* sp. | Norfolk 2 | | CH2115 | | JCU | | 23º45’ | | 168º17’ | | 377-401 | | - | | HQ439743 | | - | | HQ439668 |
| *Placotrochides scaphula* | SS022007 | | 007-014 | | CSIRO | | 44º01’50’’ | | 147º34’48’’ | | 840-1030 | | - | | HQ439744 | | - | | HQ439670 |
| *Placotrochides scaphula* | SS022007 | | 008-012 | | CSIRO | | 44º01’50’’ | | 147º34’46’’ | | 830-1030 | | - | | HQ439745 | | - | | - |
| *Truncatoflabellum australiensis* | - | | Z13194 | | WAM | | - | | - | | - | | - | | HQ439746 | | - | | HQ439671 |
| *Truncatoflabellum australiensis* | - | | Z13194 | | WAM | | - | | - | | - | | - | | HQ439747 | | - | | - |
| *Truncatoflabellum australiensis* | SS102005 | | 170-086 | | CSIRO | | 20º59’04’’ | | 114º54’25’’ | | 100-101 | | - | | HQ439748 | | - | | - |
| *Truncatoflabellum candeanum* | SS102005 | | 146-32 | | CSIRO | | 22º04’46’’ | | 113º47’45’’ | | 201-206 | | - | | HQ439749 | | - | | HQ439672 |
| *Truncatoflabellum candeanum* | SS102005 | | 153-25 | | CSIRO | | 21º59’10’’ | | 113º49’11’’ | | 165-166 | | - | | HQ439750 | | - | | - |
| *Truncatoflabellum candeanum* | SS102005 | | 096-017 | | CSIRO | | 27º48’28’’ | | 113º17’49’’ | | 112-123 | | - | | HQ439751 | | - | | - |
| *Truncatoflabellum formosum* | Norfolk 2 | | DW2137 | | JCU | | 23º01’ | | 168º23’ | | 547-560 | | - | | HQ439752 | | - | | HQ439673 |
| *Truncatoflabellum formosum* | Norfolk 2 | | DW2127 | | JCU | | 23º16’ | | 168º15’ | | 379-381 | | - | | - | | - | | HQ439674 |
| **Fungiacyathidae** | | | | | | | | | | | | | | | | | | | |
| *Fungiacyathus fragilis* | SS102005 | | 121-036 | | CSIRO | | 24º33’32’’ | | 112º15’28’’ | | 368-388 | | - | | HQ439753 | | - | | HQ439675 |
| *Fungiacyathus marenzelleri* | - | | - | | - | | - | | - | | - | | - | | EF589061 | | - | | EU262862 |
| *Fungiacyathus pusillus pacificus* | SS022007 | | 009-036 | | CSIRO | | 44º09’14’’ | | 147º07’39’’ | | 800-920 | | - | | HQ439754 | | - | | HQ439676 |
| *Fungiacyathus pusillus pacificus* | SS022007 | | 007-015 | | CSIRO | | 44º01’50’’ | | 147º34’48’’ | | 840-1030 | | - | | HQ439755 | | - | | - |
| *Fungiacyathus pusillus pacificus* | Norfolk 2 | | DW2106 | | JCU | | 23º54’ | | 167º42’ | | 685-757 | | - | | HQ439756 | | - | | - |
| *Fungiacyathus pusillus pacificus* | Norfolk 2 | | DW2106 | | JCU | | 23º54’ | | 167º42’ | | 685-757 | | - | | HQ439757 | | - | | HQ439677 |
| *Fungiacyathus stephanus* | SS102005 | | 149-019 | | CSIRO | | 22º03’34’’ | | 113º43’44’’ | | 658-754 | | - | | HQ439758 | | - | | HQ439678 |
| *Fungiacyathus turbinolioides* | SS022007 | | 008-010 | | CSIRO | | 44º01’50’’ | | 147º34’46’’ | | 830-1030 | | - | | - | | - | | HQ439679 |
| *Fungiacyathus turbinolioides* | SS022007 | | 007-015 | | CSIRO | | 44º01’50’’ | | 147º34’48’’ | | 840-1030 | | - | | HQ439759 | | - | | - |
| **Fungiidae** | | | | | | | | | | | | | | | | | | | |
| *Fungia scutaria* | - | | - | | - | | - | | - | | - | | - | | L76005 | | - | | EU262881 |
| *Zoopilus echinatus* | - | | - | | - | | - | | - | | - | | - | | L76024 | | - | | EU262870 |
| **Gardineriidae** | | | | | | | | | | | | | | | | | | | |
| *Gardineria hawaiiensis* | Bathus 4 | | DW947 | | JCU | | 20º33.72’ | | 164º57.72’ | | 470-490 | | - | | - | | - | | GQ868673 |
| *Gardineria hawaiiensis* | Norfolk 2 | | DW2057 | | JCU | | 24º40’ | | 168º39’ | | 555-565 | | GQ868660 | | GQ868701 | | GQ868678 | | - |
| *Gardineria hawaiiensis* | Norfolk 2 | | DW2060 | | JCU | | 24º40’ | | 168º39’ | | 582-600 | | GQ868657 | | GQ868702 | | GQ868677 | | - |
| *Gardineria hawaiiensis* | Norfolk 2 | | DW2084 | | JCU | | 24º52’ | | 168º22’ | | 586-730 | | GQ868658 | | GQ868699 | | GQ868679 | | - |
| *Gardineria hawaiiensis* | Norfolk 2 | | DW2126 | | JCU | | 23º16’ | | 168º14’ | | 385-550 | | GQ868659 | | GQ868687 | | GQ868680 | | - |
| *Gardineria paradoxa* | Bathus 3 | | DW784 | | JCU | | 23º56.12’ | | 169º46.14’ | | 611 | | - | | GQ868698 | | GQ868682 | | - |
| *Gardineria paradoxa* | Norfolk 2 | | DW2084 | | JCU | | 24º52’ | | 168º22’ | | 586-730 | | GQ868656 | | GQ868700 | | GQ868681 | | GQ868671 |
| *Gardineria* sp. | Norfolk 2 | | DW2136 | | JCU | | 23º01’ | | 168º23’ | | 402-410 | | - | | GQ868686 | | - | | GQ868675 |
| **Guyniidae** | | | | | | | | | | | | | | | | | | | |
| *Guynia annulata* | - | | - | | - | | - | | - | | - | | - | | AF265580 | | - | | AF549233 |
| **Meandrinidae** | | | | | | | | | | | | | | | | | | | |
| *Dichocoenia stokesi* | - | | - | | - | | - | | - | | - | | - | | AF265607 | | - | | EU262875 |
| *Meandrina meandrites* | - | | - | | - | | - | | - | | - | | - | | - | | AB117295 | | - |
| **Merulinidae** | | | | | | | | | | | | | | | | | | | |
| *Hydnophora exesa* | - | | - | | - | | - | | - | | - | | AF333059 | | - | | - | | - |
| *Hydnophora grandis* | - | | - | | - | | - | | - | | - | | - | | - | | AB117286 | | - |
| *Hydnophora rigida* | - | | - | | - | | - | | - | | - | | - | | L76009 | | - | | EU262858 |
| *Merulina ampliata* | - | | - | | - | | - | | - | | - | | AF333058 | | - | | - | | - |
| *Merulina scabricula* | - | | - | | - | | - | | - | | - | | - | | L76014 | | AB117284 | | - |
| **Micrabaciidae** | | | | | | | | | | | | | | | | | | | |
| *Letepsammia formosissima* | SS102005 | | 006-050 | | CSIRO | | 31º36’ | | 114º58’ | | 329 | | GQ868665 | | GQ868691 | | - | | GQ868668 |
| *Letepsammia formosissima* | SS102005 | | 025-020 | | CSIRO | | 35º21’ | | 118º18’ | | 398 | | GQ868663 | | GQ868697 | | GQ868685 | | GQ868672 |
| *Letepsammia formosissima* | SS102005 | | 034-055 | | CSIRO | | 35º12’ | | 118º39’ | | 431 | | GQ868664 | | GQ868696 | | GQ868684 | | GQ868670 |
| *Letepsammia formosissima* | SS102005 | | 044-066 | | CSIRO | | 35º26’ | | 118º20’ | | 900 | | GQ868662 | | GQ868692 | | - | | GQ868669 |
| *Letepsammia* sp*.* | Norfolk 1 | | DW1651 | | JCU | | 23º27.3’ | | 167º50.4’ | | 276-350 | | - | | GQ868688 | | - | | - |
| *Rhombopsammia niphada* | SS102005 | | 150-008 | | CSIRO | | 22º00’ | | 113º40’ | | 983 | | GQ868661 | | GQ868695 | | - | | - |
| *Rhombopsammia niphada* | SS102005 | | 160-016 | | CSIRO | | 21º56’ | | 113º43’ | | 1050 | | - | | GQ868693 | | GQ868683 | | GQ868674 |
| *Stephanophyllia complicate* | Bathus 4 | | CP922 | | JCU | | 18º48.04’ | | 163º18.58’ | | 600 | | - | | GQ868689 | | - | | HQ439608 |
| **Mussidae** | | | | | | | | | | | | | | | | | | | |
| *Lobophyllia hemprichii* | - | | - | | - | | - | | - | | - | | - | | L76013 | | - | | EU262833 |
| **Oculinidae** | | | | | | | | | | | | | | | | | | | |
| *Galaxea fascicularis* | - | | - | | - | | - | | - | | - | | - | | L76006 | | AB441201 | | AF263360 |
| *Madrepora oculata* | Norfolk 2 | | DW2142 | | JCU | | 23º01’ | | 168º17’ | | 550 | | - | | HQ439760 | | HM018659 | | HQ439680 |
| *Madrepora oculata* | Norfolk 2 | | DW2142 | | JCU | | 23º01’ | | 168º17’ | | 550 | | - | | HQ439761 | | - | | - |
| *Oculina patagonica* | - | | - | | - | | - | | - | | - | | - | | AF265601 | | - | | EU262842 |
| **Pectiniidae** | | | | | | | | | | | | | | | | | | | |
| *Echinophyllia orpheensis* | - | | - | | - | | - | | - | | - | | AF333065 | | - | | - | | - |
| *Mycendium elephantotus* | - | | - | | - | | - | | - | | - | | AF333057 | | - | | AB117387 | | - |
| *Mycendium* sp. | - | | - | | - | | - | | - | | - | | - | | AF265608 | | - | | EU262816 |
| **Pocilloporidae** | | | | | | | | | | | | | | | | | | | |
| *Madracis mirabilis* | - | | - | | - | | - | | - | | - | | - | | NC011160 | | - | | EU262806 |
| *Pocillopora damicornis* | - | | - | | - | | - | | - | | - | | AF333043 | | L76019 | | - | | EU262867 |
| *Pocillopora meandrina* | - | | - | | - | | - | | - | | - | | - | | L76018 | | - | | EU262803 |
| *Pocillopora verrucosa* | - | | - | | - | | - | | - | | - | | - | | - | | AB441230 | | - |
| *Stylophora pistillata* | - | | - | | - | | - | | - | | - | | - | | NC011162 | | - | | AF549253 |
| **Poritidae** | | | | | | | | | | | | | | | | | | | |
| *Porites compressa* | - | | - | | - | | - | | - | | - | | - | | L76020 | | - | | EU262814 |
| *Porites lobata* | - | | - | | - | | - | | - | | - | | - | | AF550372 | | - | | - |
| *Porites porites* | - | | - | | - | | - | | - | | - | | - | | - | | DQ643837 | | EU262878 |
| **Siderastreidae** | | | | | | | | | | | | | | | | | | | |
| *Coscinaraea* sp. | - | | - | | - | | - | | - | | - | | - | | L76001 | | - | | EU262826 |
| *Siderastrea radians* | - | | - | | - | | - | | - | | - | | - | | - | | - | | EU262861 |
| **Stenocyathidae** | | | | | | | | | | | | | | | | | | | |
| *Stenocyathus vermiformis* | Norfolk 2 | | DW2073 | | JCU | | 25º24’ | | 168º19’ | | 609 | | - | | HQ439762 | | - | | - |
| *Stenocyathus vermiformis* | ? | | ? | | CSIRO | | - | | - | | - | | - | | HQ439763 | | - | | HQ439681 |
| **Turbinoliidae** | | | | | | | | | | | | | | | | | | | |
| *Cyathotrochus pileus* | Norfolk 2 | | DW2137 | | JCU | | 23º01’ | | 168º23’ | | 547-560 | | - | | HQ439764 | | - | | HQ439682 |
| *Notocyathus* sp. | - | | - | | - | | - | | - | | - | | - | | AF265584 | | - | | EU262782 |
| *Tropidocyathus lessoni* | ? | | ? | | CSIRO | | - | | - | | - | | - | | HQ439765 | | - | | HQ439683 |
| **OUTGROUPS** | | | | | | | | | | | | | | | | | | | |
| **Actiniaria** | | | | | | | | | | | | | | | | | | | |
| *Actinia sulcata* | - | | - | | - | | - | | - | | - | | - | | - | | - | | AF549250 |
| *Anemonia* sp. | - | | - | | - | | - | | - | | - | | - | | - | | AB441274 | | - |
| *Anthosactis pearseae* | - | | - | | - | | - | | - | | - | | EU190751 | | EU190798 | | - | | EU190841 |
| *Hormosoma scotti* | - | | - | | - | | - | | - | | - | | EU190733 | | - | | - | | EU190822 |
| *Phellia gausapata* | - | | - | | - | | - | | - | | - | | - | | EU190790 | | - | | - |
| *Stichodactyla* sp. | - | | - | | - | | - | | - | | - | | - | | - | | AB441275 | | - |
| *Stomphia didemon* | - | | - | | - | | - | | - | | - | | EU190749 | | EU190795 | | - | | - |
| **Antipatharia** | | | | | | | | | | | | | | | | | | | |
| *Antipathes galapagensis* | - | | - | | - | | - | | - | | - | | - | | - | | - | | AY026365 |
| *Chrysopathes Formosa* | - | | - | | - | | - | | - | | - | | DQ304771 | | DQ304771 | | - | | - |
| *Cirripathes* sp. | - | | - | | - | | - | | - | | - | | - | | - | | AB441271 | | - |
| **Corallimorpharia** | | | | | | | | | | | | | | | | | | | |
| *Corynactis viridis* | - | | - | | - | | - | | - | | - | | - | | - | | - | | EU262836 |
| *Rhodactis mussoides* | - | | - | | - | | - | | - | | - | | AF177049 | | - | | - | | - |
| *Rhodactis rhodostoma* | - | | - | | - | | - | | - | | - | | - | | EF589054 | | - | | - |
| *Rhodactis* sp. | - | | - | | - | | - | | - | | - | | - | | - | | AB441265 | | - |
| *Ricordea florida* | - | | - | | - | | - | | - | | - | | - | | EF589057 | | - | | EU262882 |
| **Octocorallia** | | | | | | | | | | | | | | | | | | | |
| *Acanella eburnean* | - | | - | | - | | - | | - | | - | | EF672731 | | - | | EF672731 | | - |
| *Acanthogorgia* sp. | - | | - | | - | | - | | - | | - | | - | | - | | - | | FJ642927 |
| *Keratoisis* sp. | - | | - | | - | | - | | - | | - | | - | | AY351666 | | - | | - |
| **Zoanthidea** | | | | | | | | | | | | | | | | | | | |
| *Corallizoanthus tsukaharai* | - | | - | | - | | - | | - | | - | | - | | EU035626 | | - | | - |
| *Savalia savaglia* | - | | - | | - | | - | | - | | - | | DQ825686 | | - | | - | | - |
| *Zoanthus kuroshio* | - | | - | | - | | - | | - | | - | | - | | - | | AB252668 | | - |

CSIRO – Australia's Commonwealth Scientific and Industrial Research Organisation (Hobart, Australia).

JCU – James Cook University (Townsville, Australia).

WAM – Western Australian Museum (Perth, Australia).
