# Supplementary figures and images for: The ancient evolutionary origins of Scleractinia revealed by azooxanthellate corals
Source: BMC Evol Biol. 2011 Oct 28;11:316. doi: 10.1186/1471-2148-11-316 (PMC3224782; doi:10.1186/1471-2148-11-316)

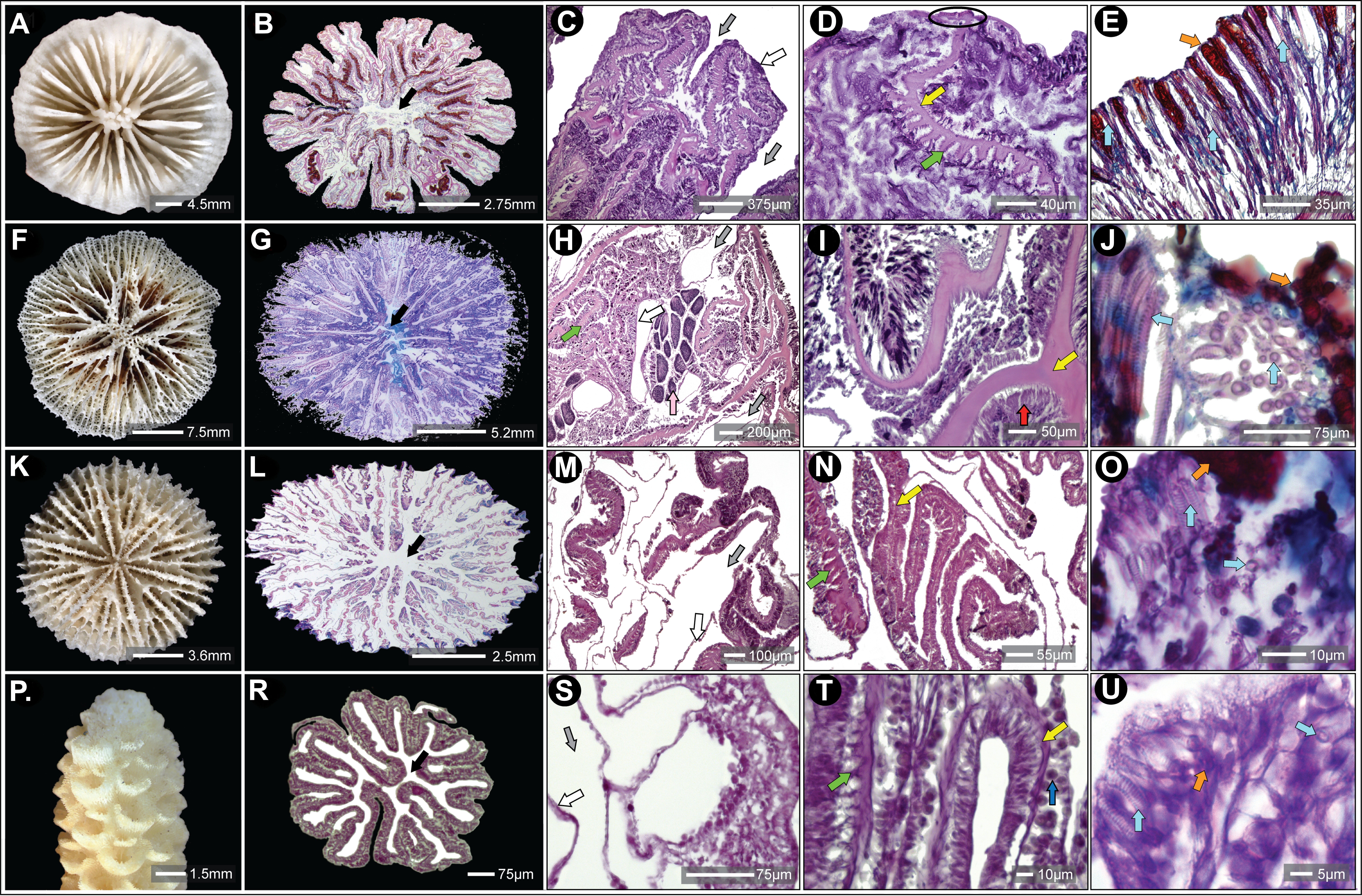

Supplement: Additional file 2 — Anatomy of Gardineria, Letepsammia and other extant scleractinian corals. The figure compares Gardineria hawaiiensis (A-E), Letepsammia formosissima (F-J), Fungiacyathus margaretae (K-O), and Acropora millepora (P-U) at the levels of skeleton macromorphology (first column), anatomy (second column) and histology (columns 3-4) (S-U, courtesy of Dr. Tracy Ainsworth). Color arrows indicate the following anatomical and histological details: black arrows, mouth/pharynx position on cross-sectioned polyps; gray arrows, septal position; pink arrows, spermaries, white arrows, calicoblastic ectoderm; yellow arrows, mesoglea; green arrows, mesogleal plates; red arrow, muscle fibers; dark blue arrows, zooxanthellae; light blue arrows, cnidae; orange arrows, mucocytes. All cross sections are stained with Alcian Blue/PAS or haematoxylin and eosin. Cnidae are shown on sections of tentacle acrospheres (E, J, O, U). Fungiacyathus margaretae and Acropora millepora were used as typical representatives of deep-water (azooxanthellate) and tropical shallow-water (zooxanthellate) Scleractinia respectively. Although the three deep-water species have significantly thicker mesoglea and mesogleal plates, and more abundant mucocytes than does the shallow water coral (A. millepora), G. hawaiiensis and L. formosissima are typical scleractinians in terms of all histological features examined. [file 1471-2148-11-316-S2.JPEG]

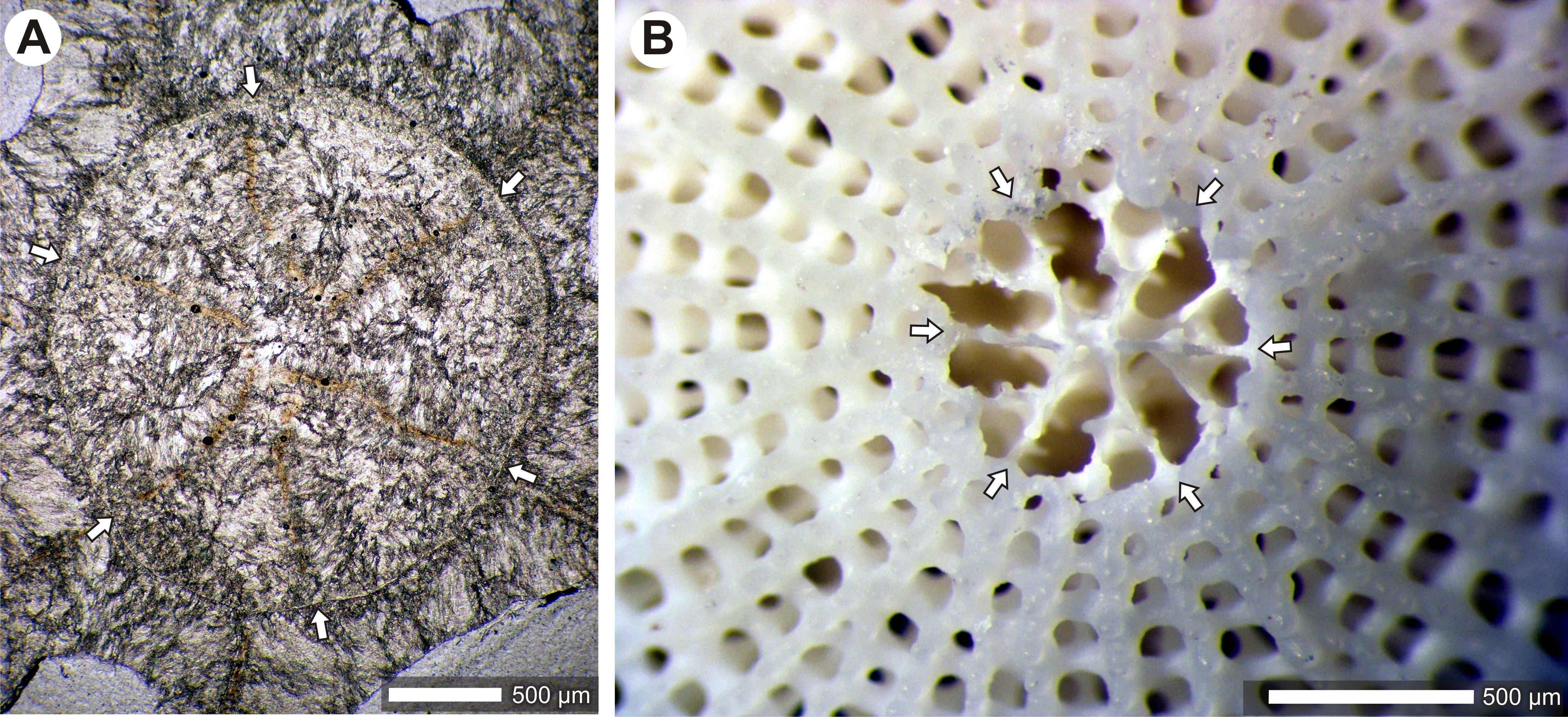

Supplement: Additional file 3 — Initial ontogenetic stages in Gardineria hawaiiensis (A) and Letepsammia formosissima (B). The position of the six simultaneously inserted protosepta are indicated with white arrows. Thin section of the corallum base (A) and polished corallum base (B). [file 1471-2148-11-316-S3.JPEG]

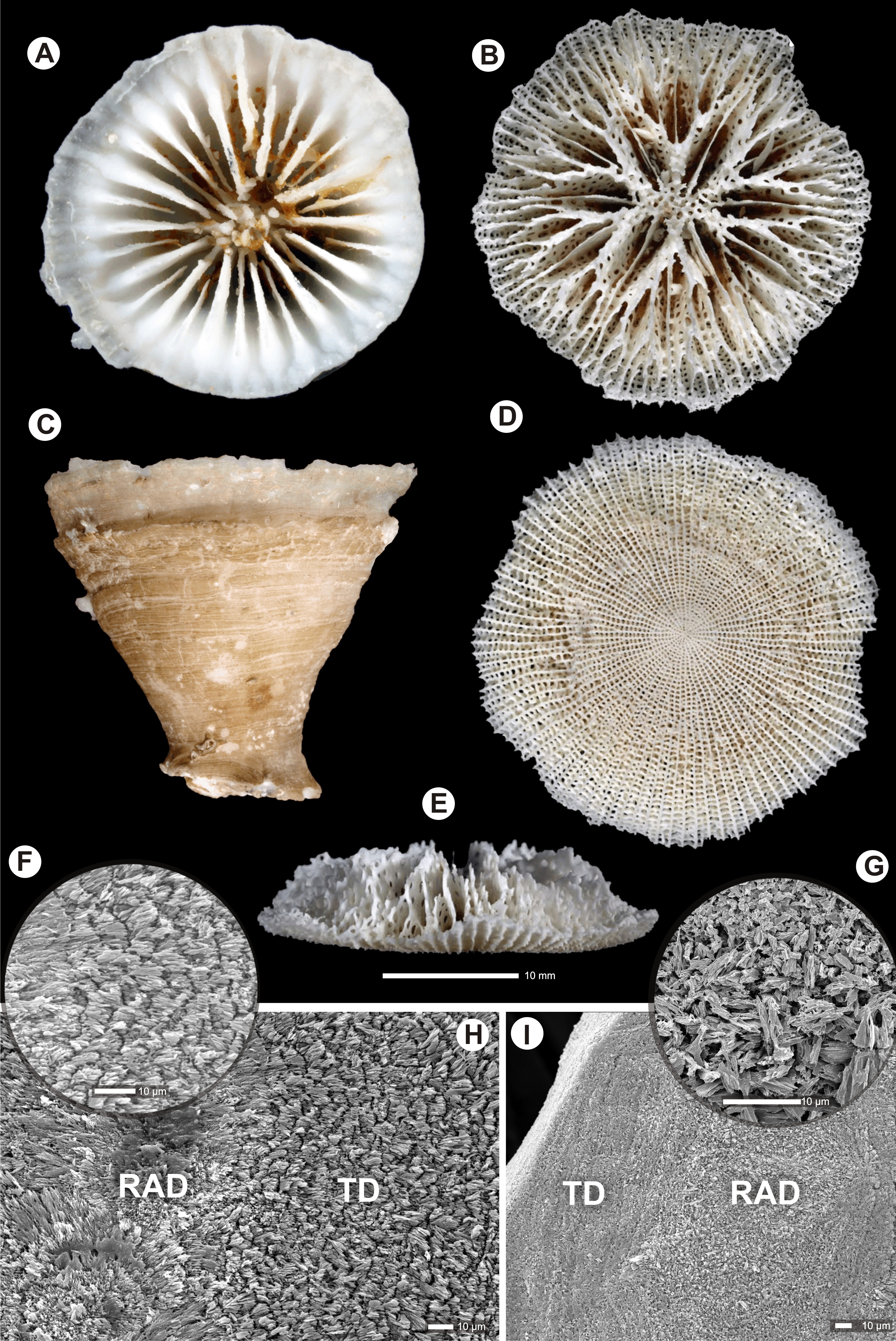

Supplement: Additional file 4 — Skeletons of modern, deep-water representatives of the Basal clade of Scleractinia: Gardineriidae (Gardineria hawaiiensis) and Micrabaciidae (Letepsammia formosissima). While gardineriids have very robust coralla (A, B), micrabaciids typically have a light, lace-like skeleton with perforated walls and septa (B, D, E). Such lightly calcified skeletons are common in corals living close to or below the carbonate compensation depth (4500-5000 m; see also Additional file 6). In addition, uniquely amongst extant corals, the thickening deposits of micrabaciids are composed of a meshwork of short and extremely thin (ca. 100-300 nm) fibers with variable crystallographic orientation (G, I). In the case of gardineriids, distinctively from most deep-water scleractinians, which display aragonite fibers in large bundles (e.g., Desmophyllum) or in complex patterns (e.g., Flabellum), septal microstructure typically forms smaller, vesicular units (F, H, see also Additional file 6). The cyclical insertion pattern of septa in gardineriids (A) and micrabaciids (B) is typical of Scleractinia. However, both taxa show several unique features that distinguish them from other modern corals and from one another. In Gardineria (C) the outer part of the skeleton consists of a thick epithecal wall, which is unique to modern corals but was common among the earliest solitary anthozoans. In contrast, the synapticular wall of micrabaciids is highly porous (D). Unique features of modern micrabaciids are the multiple bifurcations of septa of the third order, straight and non-bifurcate septa of the first order (B), and thickening deposits (TD) composed of irregular meshwork of short fibers organized into small bundles (G, I). In contrast, a central line of well-organized rapid accretion centers and radiating bundles of fibers, formed by sequentially addition of micrometer-sized growth layers characterize Gardineria septal microstructure (F, H). Distal (A, B), proximal (D), and lateral (C, E) views are [file 1471-2148-11-316-S4.JPEG]

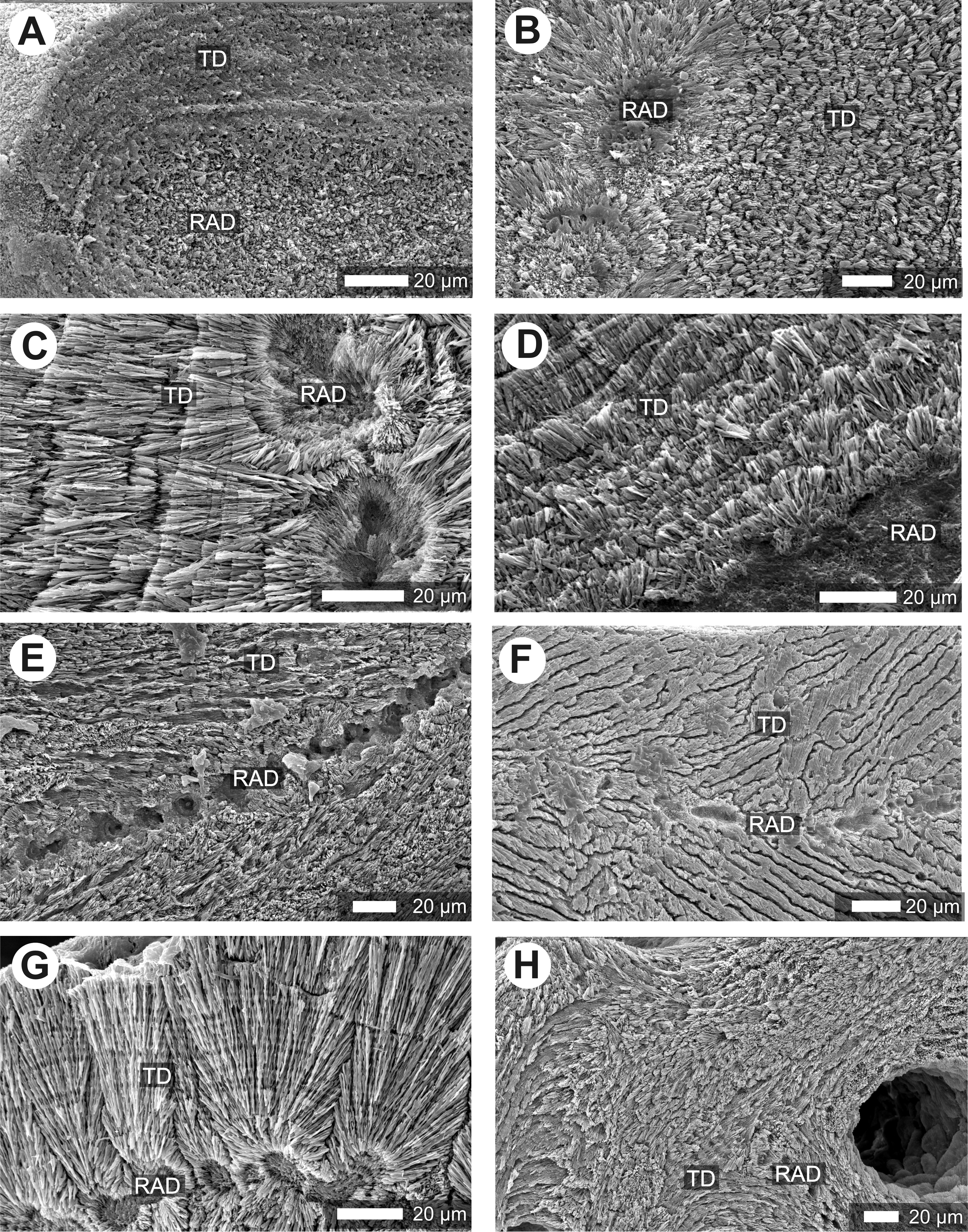

Supplement: Additional file 5 — Microstructural features of Letepsammia (Micrabaciidae), Gardineria (Gardineriidae) and other Recent scleractinian corals. The SEM micrographs shown are of etched polished surfaces of septa. In addition to differences in the distribution of Rapid Accretion Deposits (RAD), major differences can also be seen in the arrangement of the thickening deposits (TD). In Letepsammia formosissima (A), the TDs are composed of an irregular meshwork of fiber bundles oriented sub-parallel to the surface, whereas in Gardineria hawaiiensis (B), bundles of fibers (TD) form smaller, vesicular units. In Desmophyllum dianthus (C), Caryophyllia cyathus (D) and Favia stelligera (G), the TDs consist of bundles of fibers running perpendicular to the skeletal surface (in the case of the zooxanthellate coral Favia, these display high regularity, corresponding to daily growth increments). The TDs in Flabellum (E), Galaxea (F), and Acropora (H) show micro-laminar organization corresponding to the scale-like micro-texture of their skeleton surfaces. [file 1471-2148-11-316-S5.JPEG]

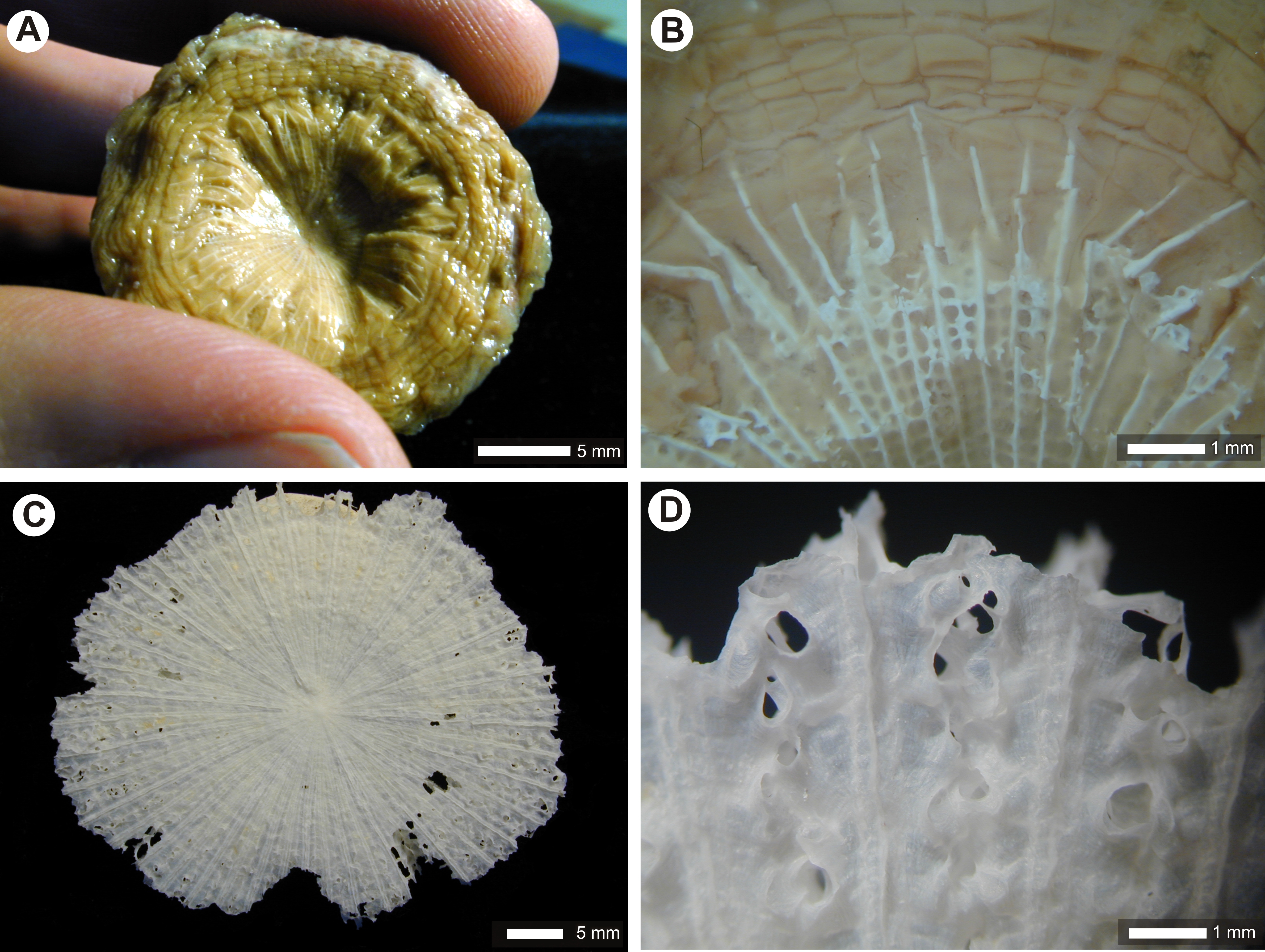

Supplement: Additional file 6 — Abyssal scleractinians. Of known scleractinians, representatives of Leptopenus (A, B) and Fungiacyathus (C, D) occur at the greatest depths (reaching depths > 5000), consequently developing fragile and thin skeletons of low density. The upper two images (A, B) are of a formaldehyde preserved specimen of Leptopenus, the bulk of the animal being composed of soft tissue (brown); the delicate skeleton (white) is deeply embedded within the polyp tissue. The two lower images (C, D) show the extremely thin, parchment-like skeleton of Fungiacyathus. Proximal views. [file 1471-2148-11-316-S6.JPEG]
